# Supplementary material for: A cluster randomised trial of a Needs Assessment Tool for adult Cancer patients and their carers (NAT-C) in primary care: A feasibility study
Source: PLoS One. 2021 Jan 28;16(1):e0245647. doi: 10.1371/journal.pone.0245647 (PMC7842977; doi:10.1371/journal.pone.0245647)
Supplement: S1 File — (DOCX) [file pone.0245647.s001.docx]

**Supporting File 1.**

| **Illustrative questions from interview guides: GP practice staff, Patients and Carers** |
| --- |
| How have you found the care you have had from your GP practice since the cancer?  Did you attend the trial needs assessment appointment? If yes, what did you think about how it compared with the usual appointments/visits?  What has been your experience of taking part in the trial? Has there been anything positive about them taking part? Any concerns?  From your experience, if we were to run a bigger version of the trial, what advice would you give us about how best to do it?  Do you feel that study training sessions (face to face / online) enabled you to effectively take part in the trial?  How has the requirement to provide a 20 minute needs assessment appointment fitted in with usual practice?  What has been your experience of implementing the NAT-C within your practice? |
