# Supplementary material for: A cluster randomised trial of a Needs Assessment Tool for adult Cancer patients and their carers (NAT-C) in primary care: A feasibility study
Source: PLoS One. 2021 Jan 28;16(1):e0245647. doi: 10.1371/journal.pone.0245647 (PMC7842977; doi:10.1371/journal.pone.0245647)
Supplement: S2 File — (DOCX) [file pone.0245647.s002.docx]

**Supporting File 2.** **Baseline** **Characteristics of patient participants with and without follow-up (missing data).**

|  | **Month 1** | | | | **Month 3** | | | | | **Month 6** | | |
| --- | --- | --- | --- | --- | --- | --- | --- | --- | --- | --- | --- | --- |
|  | **With follow-up (n=44)** | | **Without follow-up (n=3)** | | **With follow-up (n=38)** | | **Without follow-up (n=9)** | | | **With follow-up (n=32)** | | **Without follow-up (n=15)** |
| **Age**  Mean (SD)  Median (range) | 70.1 (12.1)  71 (34,88) | | 68.0 (20.1)  70 (47,87) | | 68.7 (12.3)  70 (34,88) | | 75.2 (12.5)  78 (47,48) | | | 67.8 (12.9)  69 (54,88) | | 74.6 (10.5)  78 (41,88) |
| **Sex**  Male  Female | 31 (70.5%)  13 (29.5%) | | 1 (33.3%)  2 (66.7%) | | 27 (71.1%)  11 (28.9%) | | 5 (55.6%)  4 (44.4%) | | | 22 (68.8%)  10 (31.3%) | | 10 (66.7%)  5 (33.3%) |
| **Extent of cancer**  Loco-regional  Metastatic | 36 (81.8%)  8 (18.2%) | | 2 (66.7%)  1 (33.3%) | | 31 (81.6%)  7 (18.4%) | | 7 (77.8%)  2 (22.2%) | | | 26 (81.3%)  6 (18.8%) | | 12 (80%)  3 (20%) |
| **Charlson Co-morbidity Index**  Mean (SD)  Median (range) | 1.1 (1.6)  0.0 (0,7) | | 0.3 (0.6)  0.0 (0,1) | | 1.1 (1.7)  0.0 (0,7) | | 1.1 (1.2)  1.0 (0,3) | | | 1.1 (1.7)  0.0 (0,7) | | 1.0 (1.1)  1.0 (0,3) |
| **Supportive Care Needs Survey (Total Need)**  **(a low score = a better health outcome)** | | | | | | | | | | | | |
| Mean (SD)  95% confidence intervals  Median (range)  Unmet need  No need  Missing items | 73.2 (23.5)  66.3, 80.1  68 (35,126)  31 (70.5%)  13 (29.5%)  0 | | 118.3 (42.0)  70.8, 165.8  129 (72,154)  3 (100%)  0 (0%)  0 | | 70.6 (22.7)  63.4, 77.8  65 (35,126)  25 (65.8%)  13 (34.2%)  0 | | 99.2 (31.50)  78.6, 119.8  97 (48,154)  9 (100%)  0 (0%)  0 | | | 70.8 (24.2)  62.4, 79.2  63 (35,126)  19 (59.4%)  13 (40.6%)  0 | | 87.3 (29.4)  72.4, 102.2  81 (48,154)  15 (100%)  0 (0%)  0 |
| **ESAS-r**  **(a low score = a better health outcome)** | | | | | | | | | | | | |
| Mean (SD)  95% confidence intervals  Median (range)  Missing items | 1.71 (1.70)  1.2, 2.2  1.00 (0,5.89)  1 | 3.89 (0.63)  0.0, 47.5  3.89 (3.4,4.3)  1 | | 1.65 (1.72)  1.1, 2.2  1.00 (0,5.89)  0 | | 2.65 (1.62)  1.4, 3.9  1.00 (0,5.89)  2 | | | 1.54 (1.56)  1.0, 2.1  1.00 (0,5.89)  0 | | 2.46 (1.99)  1.4, 3.5  1.89 (0,5.89)  2 | |
| **EORTC QLQ-C15-PAL**  **(a low score = a better health outcome)** | | | | | | | | | | | | |
| **Physical function**  Mean (SD)  95% confidence intervals  Median (range)  Missing items | 59.2 (32.0)  50.0, 68.7  73.3 (0.0,93.3)  0 | 35.6 (31.5)  0.0, 71.2  46.7 (0.0,60.0)  0 | | 64.2 (30.4)  54.5, 73.9  73.3 (0.0,93.3)  0 | | 30.4 (25.4)  13.8, 47.0  26.7 (0.0,73.3)  0 | | | 67.3 (30.4)  56.8, 77.8  73.3 (0.0,93.3)  0 | | 37.3 (26.5)  23.9, 50.7  33.3 (0.0,73.3)  0 | |
| **Emotional function**  Mean (SD)  95% confidence intervals Median (range)  Missing items | 80.8 (20.8)  74.7, 86.9  83.3 (41.7,100.0)  0 | 33.3 (57.7)  0.0, 98.6  0.0,100.0)  0 | | 80.9 (21.6)  74.0, 87.8  83.3 (41.7,100.0)  0 | | 64.8 (39.5)  39.0, 90.6  66.7 (0.0,100.0)  0 | | | 80.0 (21.7)  72.5, 87.5  83.3 (41.7,100.0)  0 | | 73.3 (34.4)  55.9, 90.7  83.3 (0.0,100.0)  0 | |
| **Quality of life**  Mean (SD)  95% confidence intervals  Median (range)  Missing items | 64.0 (25.7)  56.4, 71.6  66.7 (0.0,100.0)  0 | 61.1 (38.5)  17.5, 104.7  83.3 (16.7,83.3)  0 | | 63.6 (27.4)  54.9, 72.3  66.7 (0.0,100.0)  0 | | 64.8 (21.2)  51.0, 78.7  66.7 (16.7,83.3)  0 | | | 65.6 (27.7)  56.0, 75.2  66.7 (0.0,100.0)  0 | | 66.7 (22.5)  55.3, 78.1  66.7 (16.7,83.3)  0 | |
| **Fatigue**  Mean (SD)  95% confidence intervals Median (range)  Missing items | 33.1 (23.1)  26.6, 40.0  33.3 (0.0,100.0)  1 | 77.8 (29.4)  44.5, 111.1  88.9 (44.4,100.0)  0 | | 32.5 (24.3)  24.8, 40.2  33.3 (0.0,100.0)  0 | | 52.8 (27.1)  34.0, 71.6  38.9 (33.3,100.0)  1 | | | 32.3 (26.1)  22.3, 41.3  33.3 (0.0,100.0)  0 | | 44.4 (23.5)  32.1, 56.7  33.3 (22.2,100.0)  1 | |
| **Nausea**  Mean (SD)  95% confidence intervals  Median (range)  Missing items | 1.9 (5.4)  0.3, 3.5  0.0 (0.0,16.7)  0 | 38.9 (53.6)  0.0, 99.6  16.7 (0.0,100.0)  0 | | 1.3 (1.6)  0.8, 1.8  0.0 (0.0,16.7)  0 | | 16.7 (32.3)  0.0, 37.8  0.0 (0.0,100.0)  0 | | | 1.0 (4.1)  0.0, 2.4  0.0 (0.0,16.7)  0 | | 11.1 (25.7)  0.0, 24.1  0.0 (0.0,100.0)  0 | |
| **Pain**  Mean (SD)  95% confidence intervals  Median (range)  Missing items | 25.8 (31.0)  16.6, 35.0  16.7 (0.0,100.0)  0 | 66.7 (57.7)  1.4, 132.0  100.0 (0.0,100.0)  0 | | 22.8 (29.4)  13.5, 32.1  16.7 (0.0,100.0)  0 | | 51.6 (42.9)  23.6, 79.7  66.7 (0.0,100.0)  0 | | | 21.9 (29.2)  11.8, 32.0  16.7 (0.0,100.0)  0 | | 42.2 (39.8)  22.1, 62.3  33.3 (0.0,100.0)  0 | |
| **Dyspnoea**  Mean (SD)  95% confidence intervals  Median (range)  Missing items | 23.5 (30.1)  14.6, 32.4  0.0 (0.0,100.0)  0 | 22.2 (38.5)  0.0, 65.8  0.0 (0.0,66.7)  0 | | 21.1 (30.4)  11.4, 30.8  0.0 (0.0,100.0)  0 | | 33.3 (28.9)  14.4, 52.1  33.3 (0.0,66.7)  0 | | | 20.8 (30.2)  10.3, 31.2  0.0 (0.0,100.0)  0 | | 28.9 (30.5)  13.5, 44.3  33.3 (0.0,66.7)  0 | |
| **Insomnia**  Mean (SD)  95% confidence intervals  Median (range)  Missing items | 33.3 (36.7)  22.5, 44.1  33.3 (0.0,100.0)  0 | 66.7 (57.7)  1.4, 132.0  100.0 (0.0,100.0)  0 | | 38.1 (36.3)  26.5, 49.7  33.3 (0.0,100.0)  0 | | 37.0 (48.4)  5.4, 68.6  0.0 (0.0,100.0)  0 | | | 35.1 (37.8)  22.0, 48.2  33.3 (0.0,100.0)  0 | | 35.6 (40.8)  15.0, 56.2  33.3 (0.0,100.0)  0 | |
| **Appetite loss**  Mean (SD)  95% confidence intervals  Median (range)  Missing items | 15.2 (30.9)  6.0, 24.3  0.0 (0.0,100.0)  0 | 22.2 (38.5)  0.0, 65.8  0.0 (0.0,66.7)  0 | | 10.5 (29.1)  1.2, 19.8  0.0 (0.0,100.0)  0 | | 37.0 (30.9)  16.8, 57.2  33.3 (0.0,66.7)  0 | | | 8.3 (26.8)  0.0, 17.6  0.0 (0.0,100.0)  0 | | 31.1 (34.4)  13.7, 48.5  33.3 (0.0,100.0)  0 | |
| **Constipation**  Mean (SD)  95% confidence intervals  Median (range)  Missing items | 11.4 (23.8)  4.4, 18.4  0.0 (0.0,100.0)  0 | 66.7 (57.7)  1.4, 132.0  100.0 (0.0,100.0)  0 | | 8.8 (20.0)  2.4, 15.2  0.0 (0.0,66.7)  0 | | 40.7 (46.5)  10.3, 71.1  33.3 (0.0,100.0)  0 | | | 8.3 (20.7)  1.1, 15.5  0.0 (0.0,66.7)  0 | | 28.9 (40.0)  8.7, 49.1  0.0 (0.0,100.0)  0 | |
| **EQ-5D**  **(a low score = a better health outcome)** | | | | | | | | | | | | |
| Mean (SD)  95% confidence intervals  Median (range)  Missing items | 0.7 (0.2)  0.6, 0.8  0.8 (0.0,1.0)  0 | 0.7 (0.2)  0.5, 0.9  0.6 (0.6,0.9)  0 | | 0.8 (0.2)  0.7, 0.9  0.8 (0.3,1.0)  0 | | 0.5 (0.3)  0.3, 0.7  0.6 (0.0,0.9)  0 | | | 0.7 (0.2)  0.4, 1.0  0.8 (0.3,1.0)  0 | | 0.6 (0.2)  0.5, 0.7  0.7 (0.0,0.9)  0 | |
| **EQ-5D Visual Analog Scale**  **(a high score = a better health outcome)** | | | | | | | | | | | | |
| Mean (SD)  95% confidence intervals  Median (range)  Missing items | 69.6 (20.2)  64.0, 75.9  70.0 (25,100)  0 | 48.3 (33.3)  10.6, 86.0  40.0 (20,85)  0 | | 69.2 (20.7)  62.3, 75.8  70.0 (25,100)  0 | | 63.9 (24.7)  47.8, 80.0  75.0 (20,100)  0 | | | 70.0 (19.3)  63.3, 76.7  70.0 (30,100)  0 | | 60.1 (24.1)  47.9, 72.3  70.0 (20,100)  0 | |
| **ICECAP-SCM tariffs**  **(one = full capability at end of life, 0 = no capability at the end of life.** | | | | | | | | | | | | |
| Mean (SD)  95% confidence intervals  Median (range)  Missing items | 0.8 (0.1)  0.8, 0.8  0.9 (0.5,0.9)  1 | X (X)  X, X  X (X,X)  3 | | 0.8 (0.1)  0.8, 0.8  0.9 (0.5,0.9)  2 | | 0.8 (0.6)  0.4, 1.2  0.8 (0.8,0.9)  2 | | | 0.8 (0.1)  0.8, 0.8  0.9 (0.5, 0.9)  1 | | 0.8 (0.4)  0.6, 1.0  0.8 (0.7, 0.9)  3 | |
|  | | | | | | | | | | | | |
|  | **With follow-up (n=44)** | **Without follow-up (n=3)** | | **With follow-up (n=38)** | | **Without follow-up (n=9)** | | **With follow-up (n=32)** | | | **Without follow-up (n=15)** | |
| **Resource Use Questionnaire** | | | | | | | | | | | | |
| GP attendance  Yes  No  Missing items | 27 (64%)  15 (36%)  2 | 3 (100%)  0 (0%)  0 | | 23 (64%)  13 (36%)  2 | | 7 (78%)  2 (22%)  0 | | 19 (63%)  11 (37%)  2 | | | 11 (73%)  4 (27%)  0 | |
| If GP attended…  Mean attendances (SD)  95% confidence intervals  Median (range)  Missing items | 1.4 (0.8)  1.1, 1.7  1.0 (1,4)  3 | 1.7 (1.2)  0.3, 3.1  1.0 (1,3)  0 | | 1.2 (0.5)  1.0, 1.4  1.0 (1,3)  2 | | 2.2 (0.5)  1.8, 2.6  2.0 (1,4)  1 | | 1.2 (0.7)  0.9, 1.5  1.0 (1,3)  2 | | | 1.7 (1.2)  1.0, 2.4  1.0 (1,4)  1 | |
| GP home visit  Yes  No  Missing items | 3 (7%)  39 (93%)  2 | 0 (0%)  3 (100%)  0 | | 3 (8%)  33 (92%)  2 | | 0 (0%)  9 (100%)  0 | | 3 (10%)  27 (90%)  2 | | | 0 (0%)  15 (100%)  0 | |
| If GP home visit…  Mean visits (SD)  95% confidence intervals  Median (range)  Missing items | 1 (0.0)  1.0, 1.0  1 (1)  0 | NA  NA  NA  NA | | 1 (.00)  1.0, 1.0  1 (1)  0 | | NA  NA  NA  NA | | 1 (.00)  1.0, 1.0  1 (1)  0 | | | NA  NA  NA  NA | |
| District nurse visit  Yes  No  Missing items | 5 (12%)  37 (88%)  2 | 1 (33%)  2 (67%)  0 | | 3 (8%)  33 (92%)  2 | | 3 (33%)  6 (67)  0 | | 3 (10%)  27 (90%)  2 | | | 3 (20%)  12 (80%)  0 | |
| If district nurse visit…  Mean visits (SD)  95% confidence intervals  Median (range)  Missing items | 2.6 (1.5)  25.2, 27.8  3.0 (1,4)  0 | 16 (0.0)  16.0, 16.0  16 (16)  0 | | 2.7 (1.5)  1.0, 4.4  3.00 (1,4)  0 | | 7.0 (7.9)  0.0, 15.9  4.0 (1,2)  0 | | 2.7 (1.5)  1.0, 4.4  3.0 (1,4)  0 | | | 7.0 (7.9)  0.0, 15.9  4.0 (1,2)  0 | |
| Hospital admission  Yes  No  Missing items | 4 (10%)  38 (91%)  2 | 0 (0%)  3 (100%)  0 | | 3 (8%)  34 (92%)  1 | | 1 (13%)  7 (88%)  1 | | 3 (10%)  28 (90%)  1 | | | 1 (7%)  13 (93%)  1 | |
| If hospital admission, length of stay…  Mean (SD)  95% confidence intervals  Median (range)  Missing items | 8.3 (4.8)  3.6, 13.0  7.0 (4,15)  0 | NA  NA  NA  NA | | 9.0 (5.6)  2.7, 15.3  8.0 (4,15)  0 | | 6.0 (0.0)  6.0, 6.0  6 (6)  0 | | 9.0 (5.6)  2.7, 15.3  8.0 (4,15)  0 | | | 6.0 (0.0)  6.0, 6.0  6 (6)  0 | |
