# Supplementary material for: A cluster randomised trial of a Needs Assessment Tool for adult Cancer patients and their carers (NAT-C) in primary care: A feasibility study
Source: PLoS One. 2021 Jan 28;16(1):e0245647. doi: 10.1371/journal.pone.0245647 (PMC7842977; doi:10.1371/journal.pone.0245647)
Supplement: S3 File — (DOCX) [file pone.0245647.s003.docx]

**Supporting File 3.** **Supportive Care Needs Survey Contingency Table.**

| **Total needs** |  | Baseline | | |
| --- | --- | --- | --- | --- |
|  |  | No to low need | Moderate to high need | Total |
| 1 month (n=44) | No to low need | 10 (23%) | 16 (36%) | 26 (59%) |
|  | Moderate to high need | 3 (7%) | 15 (34%) | 18 (41%) |
|  | Total | 13 (30%) | 31 (70%) | 44 (100%) |
|  | *Lost to follow-up* |  | *3* | *3* |
| 3 month (n=38) | No to low need | 5 (13%) | 14 (37%) | 19 (50%) |
|  | Moderate to high need | 7 (18%) | 12 (32%) | 19 (50%) |
|  | Total | 12 (32%) | 26 (68%) | 38 (100%) |
|  | *Lost to follow-up* |  | *9* | *9* |
| 6 month  (n=32) | No to low need | 5 (16%) | 11 (34%) | 16 (50%) |
|  | Moderate to high need | 5 (16%) | 11 (34%) | 16 (50%) |
|  | Total | 10 (31%) | 22 (69%) | 32 (100%) |
|  | *Lost to follow-up* |  | *15* | *15* |

*% out of N at each time point
