# Supplementary material for: A cluster randomised trial of a Needs Assessment Tool for adult Cancer patients and their carers (NAT-C) in primary care: A feasibility study
Source: PLoS One. 2021 Jan 28;16(1):e0245647. doi: 10.1371/journal.pone.0245647 (PMC7842977; doi:10.1371/journal.pone.0245647)
Supplement: S4 File — (DOCX) [file pone.0245647.s004.docx]

**Supporting File 4.** **Change in mean score compared to baseline on the Supportive Care Needs Survey at each time point.**

|  | **1 month**  **N=44** | **3 months**  **N=38** | **6 months**  **N=32** |
| --- | --- | --- | --- |
| **Psychological needs** |  |  |  |
| Mean (SD) | -0.9 (6.7) | -1.0 (9.1) | 0.3 (7.5) |
| Median (Range) | -1 (-22,13) | 0 (-28,13) | 0 (-17,23) |
| Missing items | 0 | 2 | 0 |
| **Health system and information** |  |  |  |
| Mean (SD) | -3.3 (7.5) | -3.3 (8.8) | -4.8 (7.9) |
| Median (Range) | -1 (-24,17) | -3 (-24,15) | -5 (-22,9) |
| Missing items | 1 | 0 | 0 |
| **Physical and daily living needs** |  |  |  |
| Mean (SD) | -0.7 (3.9) | -0.5 (4.7) | 0.4 (3.7) |
| Median (Range) | 0 (-13,10) | -1 (-13,10) | 0 (-7,9) |
| Missing items | 0 | 2 | 0 |
| **Patient care and support needs** |  |  |  |
| Mean (SD) | -0.7 (4.2) | -0.8 (3.8) | -0.9 (3.7) |
| Median (Range) | 0 (-9,8) | -1 (-8,9) | -1 (-7,10) |
| Missing items | 1 | 2 | 0 |
| **Sexuality needs** |  |  |  |
| Mean (SD) | -0.6 (2.19) | -0.8 (2.6) | -0.8 (2.0) |
| Median (Range) | 0 (-6,6) | 0 (-7,3) | 0 (-5,3) |
| Missing items | 2 | 4 | 2 |
