# Supplementary material for: A cluster randomised trial of a Needs Assessment Tool for adult Cancer patients and their carers (NAT-C) in primary care: A feasibility study
Source: PLoS One. 2021 Jan 28;16(1):e0245647. doi: 10.1371/journal.pone.0245647 (PMC7842977; doi:10.1371/journal.pone.0245647)
Supplement: S5 File — (DOCX) [file pone.0245647.s005.docx]

**Supporting File 5: Summary scores of secondary outcome measures.**

| **Carer Measures** | | | | |
| --- | --- | --- | --- | --- |
|  | **Baseline (N=17)** | **1 month (N=17)** | **3 month (N=14)** | **6 month (N=10)** |
| **Carer Support Needs Assessment Tool** |  |  |  |  |
| No to low need | 11 (64.7%) | 13 (81.3%) | 12 (85.8%) | 8 (80.0%) |
| Moderate to high need | 6 (35.3%) | 3 (18.8%) | 2 (14.2%) | 2 (20.0%) |
| Missing needs | 0 | 1 | 0 | 0 |
| Mean (SD) | 17.6 (3.91) | 16.6 (3.1) | 18.2 (6.2) | 17.6 (3.7) |
| 95% confidence intervals | 15.7, 19.5 | 15.1, 18.1 | 15.0, 21.5 | 15.2, 12.0 |
| Median (Range) | 17 (14,29) | 16 (14,23) | 17 (14, 38) | 16 (14-23) |
| Missing items | 0 | 1 | 0 | 1 |
| **Carer Experience Survey (tariffs)** |  |  |  |  |
| Mean (SD) | 61.6 (17.6) | 77.5 (11.9) | 79.1 (11.8) | 75.8. (8.9) |
| 95% confidence intervals | 53.2, 70.0 | 71.0, 84.0 | 72.7, 85.5 | 70.0, 81.6 |
| Median (Range) | 63.6 (32.4,100) | 78.4 (56.3,100) | 83.5 (43.7,100) | 74.5 (63.5, 93.6) |
| Missing items | 0 | 4 | 1 | 1 |
| **Patient measures** | | | | |
|  | Baseline (N=47) | 1 month (N=44) | 3 month (N=38) | 6 month (N=32) |
| **ESAS-r** |  |  |  |  |
| Mean (SD) | 1.8 (1.72) | 2.0 (1.78) | 2.2 (1.85) | 2.3 (1.89) |
| 95% confidence intervals | 1.3, 2.3 | 1.4, 2.5 | 1.6, 2.8 | 1.6, 3.0 |
| Median (Range) | 1.1 (0,5.9) | 1.67 (0,6.4) | 1.56 (0,6.7) | 2.00 (0,7.7) |
| Missing items | 2 | 2 | 1 | 0 |
| **EORTC** |  |  |  |  |
| **Physical functioning** |  |  |  |  |
| Mean (SD) | 57.7 (32.2) | 63.2 (31.1) | 37.2 (28.1) | 68.7 (29.1) |
| 95% confidence intervals | 44.5, 66.9 | 54.0, 72.4 | 28.1, 46.2 | 58.6, 78.9 |
| Median (Range) | 60.0 (0.0,99.3) | 73.3 (0.0,93.3) | 73.3 (0.0,93.3) | 73.3 (0.0,93.3) |
| Missing items | 0 | 0 | 1 | 0 |
| **Emotional functioning** |  |  |  |  |
| Mean (SD) | 77.8 (26.2) | 81.4 (25.3) | 78.8 (30.0) | 77.6 (24.0) |
| 95% confidence intervals | 70.3, 85.3 | 73.9, 88.9 | 69.1, 88.5 | 69.3, 85.9 |
| Median (Range) | 83.6 (0.0,100.0) | 100.0 (16.7,100.0) | 100.0 (0.0,100.0) | 83.3 (16.7,100.0) |
| Missing items | 0 | 0 | 1 | 0 |
| **Quality of life** |  |  |  |  |
| Mean (SD) | 63.8 (26.1) | 66.7 (24.9) | 69.8 (24.2) | 65.6 (24.7) |
| 95% confidence intervals | 56.3, 71.2 | 59.3, 74.1 | 62.0, 77.6 | 52.0, 74.1 |
| Median (Range) | 66.7 (0.0,100.0) | 66.7 (0.0,100.0) | 66.7 (16.7,100.0) | 66.7 (16.7,100.0) |
| Missing items | 0 | 1 | 1 | 0 |
| **Fatigue** |  |  |  |  |
| Mean (SD) | 36.0 (25.7) | 41.2 (27.6) | 34.2 (28.6) | 37.2 (27.6) |
| 95% confidence intervals | 28.6, 43.4 | 26.0, 42.4 | 25.0, 43.4 | 27.6, 46.8 |
| Median (Range) | 33.3 (0.0,100.0) | 33.3 (0.0,100.0) | 33.3 (0.0,88.9) | 33.3 (0.0,100.0) |
| Missing items | 1 | 0 | 1 | 0 |
| **Nausea/Vomiting** |  |  |  |  |
| Mean (SD) | 4.3 (15.3) | 4.2 (9.6) | 3.6 (9.7) | 6.3 (15.1) |
| 95% confidence intervals | 0.0, 8.7 | 1.4, 7.0 | 0.5, 6.7 | 1.1, 11.5 |
| Median (Range) | 0.0 (0.0,100.0) | 0.0 (0.0,50.0) | 0.0 (0.0,50.0) | 0.0 (0.0,50.0) |
| Missing items | 0 | 0 | 1 | 0 |
| **Pain** |  |  |  |  |
| Mean (SD) | 28.4 (33.9) | 24.2 (30.9) | 20.3 (26.1) | 23.4 (27.7) |
| 95% confidence intervals | 18.7, 38.1 | 15.1, 33.3 | 11.9, 28.8 | 13.8, 33.0 |
| Median (Range) | 16.7 (0.0,100.0) | 16.7 (0.0,100.0) | 0.0 (0.0,83.3) | 8.3 (0.0,83.3) |
| Missing items | 0 | 0 | 1 | 0 |
| **Dyspnoea** |  |  |  |  |
| Mean (SD) | 23.4 (30.2) | 26.5 (29.3) | 21.6 (27.5) | 21.9 (28.8) |
| 95% confidence intervals | 14.8, 32.0 | 17.8, 35.2 | 12.7, 30.5 | 11.9, 31.9 |
| Median (Range) | 0.0 (0.0,100.0) | 33.3 (0.0, 100.0) | 0.0 (0.0,100.0) | 0.0 (0.0,100.0) |
| Missing items | 0 | 0 | 1 | 0 |
| **Insomnia** |  |  |  |  |
| Mean (SD) | 35.5 (38.3) | 33.3 (33.7) | 30.6 (30.8) | 30.2 (32.1) |
| 95% confidence intervals | 24.6, 46.5 | 22.3, 43.2 | 20.7, 40.5 | 19.1, 41.3 |
| Median (Range) | 33.3. (0.0,100) | 33.3 (0.0,100.0) | 33.3 (0.0,100.0) | 33.3 (0.0,100.0) |
| Missing items | 0 | 0 | 1 | 0 |
| **Appetite Loss** |  |  |  |  |
| Mean (SD) | 15.6 (31.0) | 13.6 (24.2) | 8.1 (16.5) | 13.5 (29.2) |
| 95% confidence intervals | 6.8, 24.5 | 6.5, 20.8 | 2.8, 13.4 | 3.9, 23.6 |
| Median (Range) | 0.0 (0.0,100.0) | 0.0 (0.0,66.7) | 0.0 (0.0,66.7) | 0.0 (0.0,100.0) |
| Missing items | 0 | 0 | 1 | 0 |
| **Constipation** |  |  |  |  |
| Mean (SD) | 14.9 (29.3) | 9.1 (22.0) | 13.5 (24.2) | 11.5 (24.8) |
| 95% confidence intervals | 6.5, 23.3 | 2.6, 15.6 | 5.7, 21.3 | 2.9, 20.1 |
| Median (Range) | 0.0 (0.0,100.0) | 0.0 (0.0,100.0) | 0.0, (0.0,100.0) | 0.0 (0.0,100.0) |
| Missing items | 0 | 0 | 1 | 0 |
| **EQ-5D** |  |  |  |  |
| Mean (SD) | 0.7 (0.2) | 0.7 (0.2) | 0.8 (0.2) | 0.8 (0.2) |
| 95% confidence intervals | 0.6, 0.8 | 0.6, 0.8 | 0.7, 0.9 | 0.7, 0.9 |
| Median (Range) | 0.8 (0.0,1.0) | 0.8 (0.2, 1.0) | 0.8 (0.2, 1.0) | 0.8 (0.3, 1.0) |
| Missing items | 0 | 2 | 0 | 0 |
| **EQ-5D Visual Analog Scale (0 = worst health, 100 = best health)** |  |  |  |  |
| Mean (SD) | 68.2 (21.4) | 67.6 (21.1) | 70.1 (24.4) | 70.5 (21.3) |
| 95% confidence intervals | 62.1, 74.3 | 61.4, 73.8 | 62.3, 77.9 | 63.1, 77.9 |
| Median (Range) | 70.0 (20,100) | 70.0 (25,100) | 72.5 (30,70) | 70.0 (30,98) |
| Missing items | 0 | 0 | 0 | 0 |
| **ICECAP-SCM (tariffs)** |  |  |  |  |
| Mean (SD) | 0.8 (0.1) | 0.8 (0.1) | 0.8 (0.1) | 0.8 (0.1) |
| 95% confidence intervals | 0.8, 0.8 | 0.8, 0.8 | 0.8, 0.8 | 0.8, 0.8 |
| Median (Range) | 0.9 (0.6,0.9) | 0.9 (0.5,0.9) | 0.9 (0.4,1) | 0.9 (0.5,0.9) |
| Missing items | 4 | 1 | 2 | 2 |
| **Resource Use Questionnaire** | Baseline (N=47) | 1 month (N=44) | 3 month (N=38) | 6 month (N=32) |
| GP attendance  Yes  No  Missing items | 30 (66.7%)  15 (33.3%)  2 | 31 (70.5%)  13 (29.5%)  0 | 21 (56.8%)  16 (43.2%)  1 | 14 (43.8%)  18 (56.3%)  0 |
| If GP attended |  |  |  |  |
| Mean attendances (SD) | 1.4 (0.84) | 1.7 (1.69) | 1.7 (0.90) | 2.1 (1.1) |
| 95% confidence intervals | 1.1, 1.7 | 1.0, 2.4 | 1.3, 2.1 | 1.5, 2.8 |
| Median (range) | 1.0 (1,4) | 1.0 (1,9) | 1.0 (1,4) | 2.0 (1,4) |
| Missing items | 3 | 8 | 3 | 3 |
|  |  |  |  |  |
| Mean attendances (SD) | 1.4 (0.84) | 1.7 (1.69) | 1.7 (0.90) | 2.1 (1.1) |
| 95% confidence intervals | 1.1, 1.7 | 1.0, 2.4 | 1.3, 2.1 | 1.5, 2.8 |
| Median (range) | 1.0 (1,4) | 1.0 (1,9) | 1.0 (1,4) | 2.0 (1,4) |
| Missing items | 3 | 8 | 3 | 3 |
| GP home visit  Yes  No  Missing items | 3 (6.7%)  42 (93.3%)  2 | 1 (2.3%)  43 (97.7%)  0 | 3 (7.9%)  35 (92.1%)  0 | 1 (3.1%)  31 (96.9%)  0 |
| If GP home visit |  |  |  |  |
| Mean attendances (SD) | 1.0 (.00) | 1.0 (.00) | 2.5 (2.12) | 1.0 (.00) |
| 95% confidence intervals | 1.0, 1.0 | 1.0, 1.0 | 0.0, 5.4 |  |
| Median (range) | 1 (1) | 1 (1) | 2.50 (1,4) | 1 (1) |
| Missing items | 0 | 0 | 1 | 0 |
| District nurse visit  Yes  No  Missing items | 6 (13.3%)  39 (86.7%)  2 | 5 (11.4%)  39 (88.6%)  0 | 6 (16.2%)  31 (83.8%)  1 | 4 (12.5%)  28 (87.5%)  0 |
| If district nurse visit | |  |  |  |
| Mean attendances (SD) | 4.8 (5.64) | 2.8 (1.50) | 3.2 (1.50) | 21.7 (33.23 |
| 95% confidence intervals | 0.3, 9.3 | 1.5, 4.1 | 1.9, 4.5 | -15.9, 59.3 |
| Median (range) | 3.5 (1, 16) | 3.0 (1, 4) | 3.0 (1, 5) | 4.0 (1, 60)** |
| Missing items | 0 | 1 | 1 | 1 |
| Hospital admission  Yes  No  Missing items | 4 (8.9%)  41 (91.1%)  2 | 3 (6.8%)  41 (93.2%)  0 | 5 (13.5%)  32 (86.5%)  1 | 2 (6.5%)  29 (93.5)  1 |
| If hospital admission, length of stay | |  |  |  |
| Mean attendances (SD) | 8.3 (0.48) | 5.5 (2.12) | 3.5 (3.00) | 5.3 (6.72) |
| 95% confidence intervals | 7.8, 8.8 | 2.6, 8.4 | 0.6, 6.4 | -4.0, 14.6 |
| Median (range) | 7.0 (4, 15) | 5.5 (4, 7) | 3.0 (1, 7) | 5.3 (1, 10) |
| Missing items | 0 | 1 | 1 | 0 |
| **Result distorted by one patient receiving daily visits | | | | |
