# Supplementary material for: A cluster randomised trial of a Needs Assessment Tool for adult Cancer patients and their carers (NAT-C) in primary care: A feasibility study
Source: PLoS One. 2021 Jan 28;16(1):e0245647. doi: 10.1371/journal.pone.0245647 (PMC7842977; doi:10.1371/journal.pone.0245647)
Supplement: S6 File — (DOCX) [file pone.0245647.s006.docx]

**Supporting File 6.** **Clinician responses to the NoMAD survey (n=28).**

|  | Not at all | 1 | 2 | 3 | | 4 | | 5 | 6 | | 7 | | 8 | 9 | | completely |
| --- | --- | --- | --- | --- | --- | --- | --- | --- | --- | --- | --- | --- | --- | --- | --- | --- |
| Do you feel that NAT:PD-C could become normal part of work? | 1 (4%) | 0 (0%) | 1 (4%) | 0 (0%) | | 0 (0%) | | 7 (25%) | 6 (21%) | | 6 (21%) | | 5 (18%) | 2 (7%) | | 0 (0%) |
|  | | | | | Strongly agree | | Agree | | | Neither agree nor disagree | | Disagree | | | Strongly disagree | |
| I can see how NAT-C differs from usual ways of working | | | | | 3 (11%) | | 14 (50%) | | | 7 (25%) | | 4 (14%) | | | 0 0%) | |
| I understand how the NAT-C affects the nature of my own work | | | | | 5 (18%) | | 18 (64%) | | | 4 (14%) | | 1 (4%) | | | 0 (0%) | |
| I can see the potential value of NAT-C for my work | | | | | 5 (18%) | | 20 (71%) | | | 3 (11%) | | 0 (0%) | | | 0 (0%) | |
| Key people may be needed to drive the NAT-C forward and get others involved | | | | | 9 (32%) | | 15 (54%) | | | 4 (14%) | | 0 (0%) | | | 0 (0%) | |
| I believe that using NAT-C would be a legitimate part of my role | | | | | 3 (11%) | | 20 (41%) | | | 5 (18%) | | 0 (0%) | | | 0 (0%) | |
| I am open to working with colleagues in new ways to use the NAT-C | | | | | 10 (36%) | | 17 (61%) | | | 1 (4%) | | 0 (0%) | | | 0 (0%) | |
| I am likely to support the use of NAT-C | | | | | 5 (18%) | | 22(79%) | | | 1 (4%) | | 0 (0%) | | | 0 (0%) | |
| I could easily integrate the NAT:PD-C into my existing work | | | | | 0 (0%) | | 14 (50%) | | | 12 (43%) | | 2 (7%) | | | 0 (0%) | |
| The NAT-C may disrupt working relationships | | | | | 1 (4) | | 3 (11%) | | | 10 (36%) | | 13 (46%) | | | 1 (4%) | |
| The training provided is sufficient to enable staff to implement the NAT-C | | | | | 4 (14%) | | 19 (68%) | | | 5 (18%) | | 0 (0%) | | | 0 (0%) | |
| Sufficient resources are available to support the use of the NAT-C | | | | | 4 (14%) | | 10 (36%) | | | 13 (46%) | | 0 (0%) | | | 1 (4%) | |
| Management will adequately support the use of the NAT-C | | | | | 3 (11%) | | 19 (68%) | | | 6 (21%) | | 0 (0%) | | | 0 (0%) | |
| Practice staff will agree that use of the NAT-C is worthwhile | | | | | 1 (4%) | | 16 (57%) | | | 11 (39%) | | 0 (0%) | | | 0 (0%) | |
| Feedback about the NAT-C could be used to improve it in the future | | | | | 7 (25%) | | 20 (71%) | | | 1 (4%) | | 0 (0%) | | | 0 (0%) | |
| I could modify how I work with the NAT-C | | | | | 2 (7%) | | 23 (82%) | | | 3 (11%) | | 0 (0%) | | | 0 (0%) | |
