# Supplementary material for: A cluster randomised trial of a Needs Assessment Tool for adult Cancer patients and their carers (NAT-C) in primary care: A feasibility study
Source: PLoS One. 2021 Jan 28;16(1):e0245647. doi: 10.1371/journal.pone.0245647 (PMC7842977; doi:10.1371/journal.pone.0245647)
Supplement: S1 Study — (DOCX) [file pone.0245647.s010.docx]

**Study protocol**

**Title**

A cluster randomised feasibility trial (cRCT) to test the routine use of the Needs Assessment Tool Progressive Disease Cancer (NAT:PD-C) in primary care to reduce unmet patient and carer needs and determine the feasibility of a definitive trial

**Short title**

CANAssess:PC - Cancer Needs Assessment in Primary Care: A feasibility study

**IRAS Number:** 221366

**ISRCTN Number / Clinical trials.gov Number:**

**Date of Protocol:** 08.05.2017

**Protocol Number:** 2.0

**Funder:**  Yorkshire Cancer Research (YNA142/7481)

**Study Sponsor**: University of Hull

**Confidentiality** Information in this protocol should not be disclosed, other than to those

**Statement:** involved in the execution or ethical review of the study, without written

authorisation from the Sponsor. Sponsor accepts no responsibility for the accuracy of content reproduced from this protocol and incorporated into additional documentation developed by collaborating or third party organisations.

**Regulatory** All study procedures will be conducted within ICH GCP guidelines and all

**Statement:** other regulatory requirements.

**Protocol** This protocol has been prepared in accordance with CONSORT guidelines

**Preparation:** and with regard to HRA guidance. It complies with Good Clinical Practice in

clinical research.

Authorised by:

Name: Prof Miriam Johnson Role: Chief Investigator

Signature:
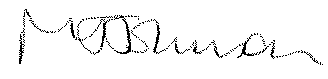
 Date: 09.02.2017

**Abstract**

**Title**: A cluster randomised feasibility trial (cRCT) to test the routine use of the Needs Assessment Tool Progressive Disease Cancer (NAT:PD-C) in primary care to reduce unmet patient and carer needs and determine the feasibility of a definitive trial

**Introduction**: This is a cluster Randomised Controlled feasibility Trial (cRCT) with embedded qualitative and process mapping studies to test the feasibility of a definitive trial of the Needs Assessment Tool – Progressive Disease Cancer (NAT: PD-C). The NAT:PD-C is a psychometrically valid needs assessment tool which has been effective in reducing unmet reported needs in Oncology clinics but has not been tested in primary care.

**Research question**: Is a cluster Randomised Controlled Trial (cRCT) to test the routine use of the NAT:PD-C in primary care to reduce unmet patient and carer needs feasible?

**Objectives**: 1) To test the feasibility and acceptability in terms of participant recruitment, uptake and delivery of training tools and electronic NAT:PD-C, completion rates and appropriateness of participant reported questionnaires at baseline, 1, 3 and 6 months 2)To identify systemic barriers and enablers of the adoption of the NAT:PD-C in to general practice.

**Intervention**: 1) Promotion and use of the NAT:PD-C with directed clinical encounter with NAT:PD-C trained clinician 2) Promotion and use of the NAT:PD-C with clinical encounter a with a clinician in line with usual practice.

**Methods:** 40-60 patients (and their carers if appropriate) with a diagnosis of active, incurable cancer will be recruited for a needs assessment consultation within a six month period from four General Practices. Participants will be assessed with regard to self-reported unmet supportive and palliative care needs at baseline, 1, 3 and 6 months. Patients will be identified through the practice cancer registry. A purposive sample of patients/carers, practice staff and clinicians from relevant referral services (e.g. specialist palliative care) will take part in interview/focus group to explore views on study procedures/measures (patients and carers) and issues regarding implementation of the NAT:PD-C.

**Outcomes/analysis:** The trial will be reported in accordance with the CONSORT 2010 statement extension to pilot and feasibly trials.^[[1]](#endnote-1)^ Descriptive statistics will be reported for the feasibility outcomes: recruitment rates by practice; intervention uptake, delivery and time from baseline measures to needs assessment appointments. Descriptive statistics for secondary outcomes will be reported to inform a potential definitive study in terms of patient/carer self-reported needs: Supportive Care Needs Survey (SCNS-SF34), Edmonton Symptom Assessment System (ESAS-r), EORTC QLQ-C15-PAL, Resouce Use Questionnaire (RUQ), Charlson Co-morbidity Index (CCI), Australian Modified Karnofsky Scale (AMKS), ICECAP Supportive Care Measure (ICECAP-SCM), EQ-5D-5L [patients]; Carer Support Needs Assessment Tool (CSNAT), Carer Experience Scale (CES) [carers]; Needs Assessment Tool: Progressive Disease Cancer ( NAT:PD-C) [practice clinicians]). Variability in these measures at both the level of patient and carer and GP practice will be calculated. Using these results, a sample size for an RCT will be estimated which will include estimation of an intraclass correlation coefficient.

Interview/focus group data will be analysed using template analysis/hierarchical coding and guided by normalisation process theory to determine the acceptability and feasibility of a definitive trial.

Recruitment will commence Spring 2017.

**Key contacts:**

**Lead Study** Professor Miriam Johnson (Chief Investigator)

**Investigator:** Professor in Palliative Medicine

Director of the Wolfson Palliative Care Research Centre

Hull York Medical School

The University of Hull

Address for correspondence:

Wolfson Palliative Care Research Centre

Supportive care, Early Diagnosis and Advanced disease (SEDA) Research

Group

Hull York Medical School, Hertford Building

University of Hull, Cottingham Road

Hull, HU6 7RX

E-mail: miriam.johnson@hyms.ac.uk

Phone: 01482 463309

Fax: 01482 464705

**Trial Manager:** Dr Joseph Clark

Research Fellow in Palliative Care

Wolfson Palliative Care Research Centre

Hull York Medical School, University of Hull

HU6 7RX

E-mail: joseph.clark@hyms.ac.uk

Phone: 01482 463123

Fax: 01482 464705

**Trial Researcher:** Dr Elvis Amoakwa

Research Associate

Wolfson Palliative Care Research Centre

Hull York Medical School, University of Hull

HU6 7RX

E-mail: elvis.amoakwa@hyms.ac.uk

Phone: 01482 463123

Fax: 01482 464705

**Trial Statistician:** Dr Victoria Allgar

Senior Lecturer in Medical Statistics

Wolfson Palliative Care Research Centre

Hull York Medical School, University of Hull

HU6 7RX

**Sponsor contact**: Dr Andrew Taylor

Research Excellence Manager

Research and Enterprise

University of Hull, Cottingham Road

HU6 7RX

Phone: 01482 465317

E-mail: A.F.Taylor@hull.ac.uk

**Investigator Team:**

| **Name** | **Title** | **Faculty/department** | **Contact details** |
| --- | --- | --- | --- |
| Prof Miriam Johnson  (Chief Investigator) | Professor of Palliative Medicine | Hull York Medical School, University of Hull | E) miriam.johnson@hyms.ac.uk  T) 01482 850850 |
| Prof Una Macleod | Professor of Primary Care Medicine | Hull York Medical School, University of Hull | E) una.macleod@hyms.ac.uk  T) 01482 463806 |
| Prof Amanda Farrin | Professor of Clinical Trials & Evaluation of Complex Interventions, CTRU | Leeds Institute of Clinical Research, University of Leeds | E) a.j.farrin@leeds.ac.uk  T) 0113 343 8017 |
| Dr David Meads | Associate Professor in Health Economics | Academic Unit of Health Economics, University of Leeds | E) d.meads@leeds.ac.uk  T) 0113 343 0860. |
| Ms Alexandra Wright-Hughes | Senior Medical Statistician, CTRU | Leeds Institute of Clinical Research, University of Leeds | E) a.wright-hughes@leeds.ac.uk  T) 0113 343 1477 |
| Prof John Blenkinsopp | Professor of Organisational Behaviour & Human Resource Management | Newcastle Business School, Northumbria University | E) john.blenkinsopp@northumbria.ac.uk  T) 0191 227 3947 |
| Prof Robbie Foy | Professor of Primary Care | Leeds Institute of Health Sciences, University of Leeds | E) r.foy@leeds.ac.uk  T) 0113 343 4879 |
| Victoria Allgar | Senior Lecturer in Medical Statistics | Hull York Medical School / Health Sciences, University of Hull, University of York Associate | E) victoria.allgar@york.ac.uk  T) 0870 1245500 |
| Prof Hamish Fraser | Professor of eHealth | Leeds Institute of Health Sciences, University of Leeds | E) h.fraser@leeds.ac.uk  T) 0113 343 6940 |

| **Protocol history** |  |  |
| --- | --- | --- |
| **Version** | **Author** | **Reason** |
| 0.1 | J Clark | New protocol from draft |
| 0.2 | J Clark | Reviewed by Chief Investigator M Johnson |
| 0.3 | J Clark | Review by TMG |
| 0.4 | J Clark | Review by study statistician (V Allgar) |
| 0.5 | J Clark | Public-patient review |

| **Protocol approval** |  |  |
| --- | --- | --- |
| **Version** | **Author** | **Reason** |
| 1.0 | J Clark |  |
| 2.0 | J Clark | Response to requested amendments from Provisional Favourable REC opinion |

**List of contents**

| **GENERAL INFORMATION** |  |
| --- | --- |
| **Title page and declarations** | 1 |
| **Research reference numbers** | 1 |
| **Abstract** | 2 |
| **Key trial contacts** | 3 |
| **Investigator team** | 4 |
| **Protocol approval history** | 5 |
| **List of contents** | 6 |
| **List of abbreviations** | 8 |
| **Study flow diagram** | 9 |
| **SECTION** |  |
| **1. Background and rationale** | 10 |
| 1.1 The Needs Assessment Tool – Progressive Disease Cancer (NAT: PD-C) | 10 |
| 1.2 Relevance to Yorkshire | 10 |
| 1.3 Preliminary work | 11 |
| 1.4 The need for a feasibility study | 11 |
| **2. Study aims and objectives** | 11 |
| 2.1 Aim of the research | 11 |
| 2.2 Study objectives | 11 |
| **3.0 Methods** | 12 |
| 3.1 Study setting | 12 |
| 3.2 Study design | 12 |
| 3.3 Pragmatic cluster Randomised Controlled feasibility Trial (cRCT) | 13 |
| 3.3.1 Eligibility criteria | 13 |
| 3.3.2 Consent procedures | 14 |
| 3.3.3 Randomisation | 15 |
| 3.3.4 Interventions | 15 |
| 3.3.5 Study assessment schedule | 16 |
| 3.3.6 Assessment Instruments | 18 |
| 3.3.7 Study feasibility outcomes | 19 |
| 3.3.8 Sample size | 20 |
| 3.4 Qualitative Evaluation methods | 21 |
| 3.4.1 Eligibility Criteria | 21 |
| 3.4.2 Sample size | 21 |
| 3.4.3 Recruitment and consent | 21 |
| 3.4.4 Qualitative data collection | 23 |
| **4.0 Data analysis** | 24 |
| 4.1 Cluster Randomised Controlled Feasibility Trial: Statistical Analysis Plan (SAP) | 24 |
| 4.2 Qualitative process evaluation | 24 |
| **5.0 Data management and confidentiality** | 25 |
| 5.1 Confidentiality | 25 |
| 5.2 Data management | 25 |
| 5.3 Data monitoring and quality assurance | 26 |
| **6.0 Ethical considerations** | 26 |
| 6.1 Safety issues | 27 |
| 6.2 Potential benefits for participants | 27 |
| 6.3 Potential risks and burdens to participants | 27 |
| 6.4 Potential risks for the study team and health professionals | 28 |
| 6.5 Risks and benefits of the study | 28 |
| 6.6 Reimbursements | 28 |
| **7.0 Study management** | 28 |
| 7.1 Trial Management Group (TMG) | 28 |
| 7.2 Core Project Team (CPT) | 29 |
| 7.3 Trial Steering Committee (TSC) | 29 |
| 7.4 Public Patient Involvement (PPI) | 29 |
| **8.0 Safety reporting** | 29 |
| 8.1 Stopping rules | 29 |
| 8.2 Definitions | 29 |
| 8.3 Progress Reports | 30 |
| **9.0 Dissemination and archiving** | 30 |
| 9.1 Dissemination plan | 30 |
| 9.2 Archiving | 30 |
| **10. Gantt chart** | 31 |
| **11. References** | 32 |
|  |  |

**List of abbreviations**

AE Adverse Event

AKPS Australian modified Karnofsky Scale

AR Adverse Reaction

CANAsssess:PC Cancer Needs Assessment Primary Care

CCG Clinical Commissioning Group

CI Chief Investigator

CIS Carer Information Sheet

CPT Core Project Team

CRN Clinical Research Network

cRCT cluster Randomised Controlled Trial

CSNAT Carer Support Needs Assessment Tool

DBS Disclosure and Barring Service

ESASr Revised Edmonton Symptom Assessment System

HREC Human Research Ethics Committee

GCP Good Clinical Practice

GP General Practitioner

ICECAP-SCM ICECAP Supportive Care Measure

MRC Medical Research Council

NAT: PD-C Needs Assessment Tool – Progressive Disease Cancer

NRES National Research Ethics Service

PCIS Patient/Carer Information Sheet

PI Principal Investigator

PIS Patient Information Sheet

PPI Public-Patient Involvement

REC Research Ethics Committee

RUQ Resource Use Questionnaire

SAE Serious Adverse Event

SAP Statistical Analysis Plan

SCNS Supportive Care Needs Survey

SEDA Supportive care, Early Diagnosis and Advanced disease

SOP Standard Operating Procedure

TMG Trial Management Group

TSC Trial Steering Committee

UK United Kingdom

YCR Yorkshire Cancer Research

**Study flow diagram**


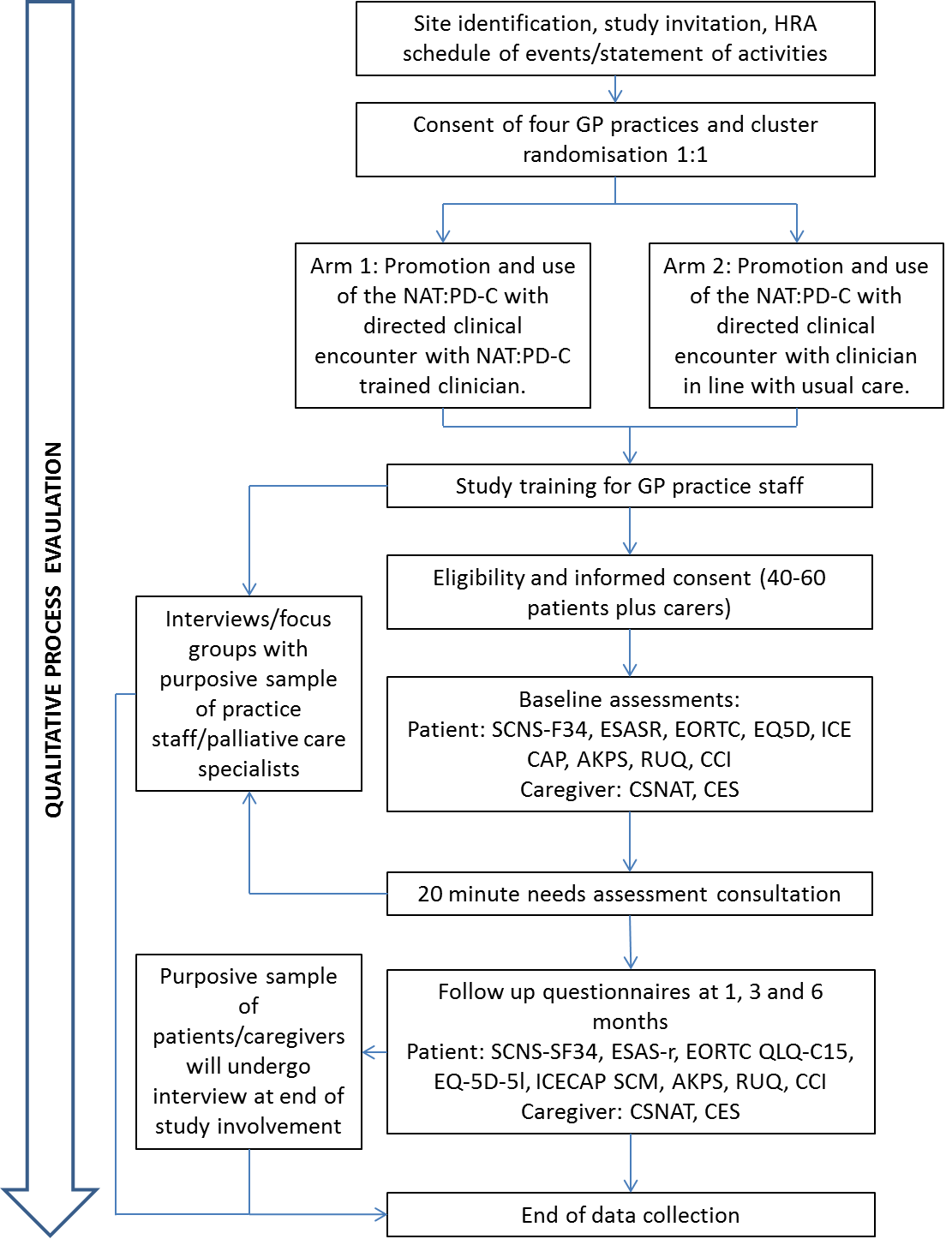
**A cluster randomised feasibility trial (cRCT) to test the routine use of the NAT:PD-C in primary care to reduce unmet patient and carer needs and determine the feasibility of a definitive trial**

**1.0 Background and rationale**

The World Oncology Forum has called for governments and policy makers to ensure that people with cancer have access to essential diagnostics, curative and palliative care.^[[2]](#endnote-2)^ However, despite this, patients still experience significant levels of unmet palliative care need, particularly within the psychological and physical and daily activity domains,^[[3]](#endnote-3)^ where around half of patients have continuing concerns.^[[4]](#endnote-4)^,^[[5]](#endnote-5)^ The 2014 National Cancer Patient Experience Survey showed that many patients felt their GP practice doctors and nurses could do more to help during their cancer treatment.^[[6]](#endnote-6)^ In addition, there are extensive needs experienced by family and close friends providing care at the end of life, ranging from practical help, information and communication, emotional and psychological support to financial and legal issues.^[[7]](#endnote-7)^,^[[8]](#endnote-8)^

There are many needs assessment tools available to assist clinicians to provide palliative care for people with cancer.^[[9]](#endnote-9)^ Yet few are designed to identify and triage palliative care needs in cancer patients in the everyday busy clinical setting. Ideally, a clinician-administered structured need assessment tooliv should prompt discussion of concerns between patients, families and health professionals, triage according to an individual’s burden of unmet need, and prioritise resources and identify service areas for improvement.^[[10]](#endnote-10),^^[[11]](#endnote-11),^^[[12]](#endnote-12)^^,^^[[13]](#endnote-13)^,^[[14]](#endnote-14)^

An assessment tool should help reduce late referrals for palliative care for people with cancer, and improve referrals where there are physical, psychological, social and spiritual problems.^[[15]](#endnote-15)^ However, the tools currently available tend to be too detailed and long for daily clinical use by the non-palliative care specialist;xiii,xiv,^[[16]](#endnote-16)^ and indeed, in the United Kingdom [UK], half of the sampled health organisations did not use any assessment tool whatsoever.^[[17]](#endnote-17)^ Furthermore, although needs assessment tools are advocated, there is no rigorous research evidence to indicate whether they actually improve practice and patient outcomes.

**1.1. The Needs Assessment Tool – Progressive Disease Cancer (NAT: PD-C)**

*The Needs Assessment Tool – Progressive Disease Cancer* (NAT: PD-C)^[[18]](#endnote-18)^ is a generic one-page psychometrically valid, reliable and clinically acceptable tool for assessment of patients’ and carers’ palliative care needs across a broad range of domains.^[[19]](#endnote-19)^ It differentiates between need that can be addressed by the usual care team and that which requires referral for specialist palliative care. It has been shown to reduce patient reported unmet need in oncology clinics.v If it were found to be helpful in the primary care assessment of palliative care cancer patients, it could readily be adapted for all patients on the cancer registry to provide a systematic approach to cancer reviews in primary care.^[[20]](#endnote-20)^

**1.2 Relevance to Yorkshire**

People living in Yorkshire have a higher death rate from cancer than England as a whole, thus excellent care at the end of life is very important. Over the past decade the number of palliative care consultant physicians in the region has increased but access to both general and specialist palliative care is still inconsistent and lags behind other areas in England.^[[21]](#endnote-21)^ This makes the NHS Hull Clinical Commissioning Group (CCG) and surrounding CCGs (e.g. NHS East Riding of Yorkshire CCG) an appropriate location for a feasibility study to test the NAT:PD-C. Should the proposed study demonstrate the feasibility of the study design in terms of recruitment, data collection, acceptability and patient outcomes, it would be appropriate to proceed to a definitive trial across a wider range of study sites and regions.

**1.3 Preliminary work**

As part of a Yorkshire Cancer Research (YCR) funded programme (Programme Principal Investigator (PI) Una Macleod, Project Chief Investigator Miriam Johnson), the study team has completed a Phase I study including: adaptation, face and content validation and inter-rater reliability testing of the NAT:PD-C for use in UK primary care of cancer patients. Agreement was tested using the Fleiss weighted kappa. Fair to moderate agreement was seen for most domains of the NAT, especially for patient needs. Agreement was higher for carer wellbeing domains than in the original NAT:PD-C testing.

Given that there was wide variation in the group of assessors, even fair to moderate level of represents a useful tool to provide systematic and standard assessment and may be enough to stimulate enough change in practice to improve outcomes. Indeed, when tested, use of the original NAT:PD-C was associated with a significant reduction in needs following consultations in terms of: information, patient care and support.v

**1.4 The need for a feasibility study**

Prior to embarking on a definitive cluster Randomised Controlled Trial (cRCT) the following uncertainties need to be addressed in relation to: a) recruitment; b) uptake and delivery and uptake of the NAT:PD-C; c) data collection and quality; d) which sub-scales on the Supportive Care Needs Survey (SCNS) are most suitable as the primary outcome for a definitive trial.

The study team has developed a computerised template for the NAT:PD-C which can be embedded within General Practitioner (GP) computer systems. A paper copy of the NAT:PD-C will also be present at all NAT-guided consultations to ensure access to the tool in the event of technological failure, or for consultations undertaken in non-practice settings, e.g. patient homes. This project will determine the feasibility of a cluster randomised trial to assess whether the use of the primary care electronic NAT:PD-C results in improved patient care, accounting for variation in the implementation and use of the tool among primary care clinicians.

**2.0 Study aims and objectives**

**2.1 Aim of the research**

To assess the feasibility of a definitive cluster Randomised Controlled Trial (cRCT) to test the routine use of the NAT:PD-C in primary care to reduce unmet patient and carer needs.

**2.2 Study objectives**

The objectives of this mixed-methods study relate to the feasibility of implementing a definitive cRCT and are situated across three domains:

a. Recruitment:

- To test the feasibility of recruiting GP practices to test the NAT:PD-C
- To test the feasibility and acceptability of the proposed participant recruitment methods to patients, carers and GP practices.

b. Uptake and delivery:

- To assess methods to conduct the index NAT:PD-C framed consultation with varying levels of pragmatism; should the patient be directed to make an appointment with a GP or nurse in line with usual practice, or with one which the study team know has completed NAT:PD-C training?
- To determine the acceptability and uptake of the training tools and electronic NAT:PD-C by GP practice staff.
- To explore the views of palliative care specialists regarding how the NAT:PD-C will work in practice with regard to potential impact on their working practices
- To identify factors influencing the willingness of patients/carers to consent to the trial, complete study measures
- To identify organisational factors which might influence the willingness or ability of GP practices to adopt the NAT:PD-C.

c. Data collection and quality:

- To assess the feasibility of methods for collection of clinical data, NAT:PD-C forms, health service utilisation and referrals
- To identify the patient and carer outcomes most relevant for the primary outcome of a definitive trial assessed by completion and follow up rates for participant reported questionnaires at baseline, 1, 3 and 6 months, the level of and patterns of missing outcome data and participant views.

**3.0 Methods**

**3.1 Study setting**

The study will be conducted in primary care. Four GP practices will be recruited from the Hull, East Ridings, North Lincolnshire and North Yorkshire Clinical Commissioning Groups (CCGs).

These areas were selected as the NAT:PD-C was ‘made in Yorkshire’ and the research team has significant experience in running studies in the Yorkshire region including recruiting from GP practices. The addition of North Lincolnshire is for pragmatic reasons based upon the advice of the Clinical Research Network (CRN) to ensure successful recruitment of GP practices.

**3.2 Study design**

This is a two-arm feasibility cluster Randomised Controlled Trial (cRCT) with a parallel qualitative evaluation involving four general practices selected and trained to use the NAT:PD-C in routine consultations for patients with active cancer. Practices will be randomised to different approaches for arranging NAT:PD-C consultations. Our mixed-methods approach has been designed to answer our research question:

Is a cluster Randomised Controlled Trial (cRCT) to test the routine use of the NAT:PD-C in primary care to reduce unmet patient and carer needs feasible?

Our mixed-methods study comprises two distinct methods in order answer our research question: a cluster Randomised Controlled feasibility Trial (cRCT) and a qualitative evaluation of training procedures and the process of implementation of NAT PD-C into general practice. The methods for each element of this mixed-methods study are presented separately below.

**3.3 Pragmatic cluster Randomised Controlled Trial (cRCT): a Feasibility Study**

**3.3.1 Eligibility criteria**

Inclusion:

Cluster level:

- 1. General practices expressing a willingness to be trained and to use the NAT:PD-C.
  2. Written informed consent provided by practice manager or deputy.

GP practices will be identified and recruited drawing upon existing contacts of the research team including physicians who contributed to the validation of the NAT:PD-C and with the assistance of the Clinical Research Network (CRN).

Patient level:

1. Adults (aged 18 and above)
2. Diagnosis of active incurable cancer
3. Willing to have a consultation with a practice clinician
4. Able to complete study measures
5. Written or observed verbal informed consent

Exclusion:

1. Patients in complete remission
2. Patients receiving treatment with intent to cure (patients receiving anti-cancer treatments with the intention to palliate, OR receiving supportive care only will be eligible).
3. Patients living in a care home or other institutional setting
4. Patients who do not speak English well enough to provide informed consent and complete study measures.
5. Known to have a co-morbid condition which means they lack sufficient mental capacity to provide informed consent in the opinion of the clinician (e.g. dementia)
6. Within one month of receiving their cancer diagnosis

Potentially eligible patients will be identified from the cancer register by a clinician or opportunistically during usual care appointments. Patients do not have to nominate a carer in order to participate in the study.

Carers:

1. Adults (aged 18 and above)
2. Nominated by the patient
3. Able to complete study measures
4. Written or observed verbal informed consent

Exclusion:

1. Carers who do not speak English well enough to provide informed consent and complete study measures.
2. Paid carers

Participating patients will be invited to nominate their primary carer who will be assessed for eligibility and invited to participate in the study. ‘Carer’ refers to those who are close to (and have been nominated by) the patient and provide support even if they are not providing personal care. The eligibility of carers will be assessed by a researcher during the initial visit and prior to informed consent being taken.

**3.3.2 Consent procedures**

Cluster:

Four practices will be recruited from Hull and surrounding areas to take part in the feasibility study. GP practices of participants who have already contributed to Phase I of the study will initially be targeted. Thereafter, GP practice managers in the Hull and surrounding area will be contacted by the research team, using existing contacts from the Phase I study and from within the Yorkshire and Humber Clinical Research Network (CRN). Potential study sites will initially be contacted by the research team with a study invitation, a Schedule of Events and / Statement of Activities.

Consent will be provided by the practice manager or deputy through the agreement of the Schedule of Events and Statement of Activities, evidenced by a contract between the GP practice and the University of Hull. Consent is given for practice involvement as stipulated by the HRA schedule of events and statement of activities. All necessary permissions and governances will be gained prior to starting research activity.

All participating GP practices will be trained by the SEDA research team in the use of the NAT:PD-C prior to study participation.

Patients and carers:

Eligible patients will be identified by a clinician by searching the practice cancer register.

Patients’ usual care team will then invite patients to participate in the study by sending a study invitation and Patient Information Sheet (PIS). A clinician will note instances where a patient usually has a translated consultation and is not sent a study invitation to inform translation provision in a full trial. From the pool of potentially eligible patients, Study Invitations will be sent out periodically to ensure that the research team and GP practices have capacity to follow study procedures. A screening log will be maintained to ensure that potential participants are not contacted more than once/twice. The cancer registry will be searched by a clinician regularly to identify new potentially eligible patients.

We will not approach patients until at least one month after they have received their diagnosis to allow them time to process this. Potentially eligible patients diagnosed within one month, will be kept on record and invited to the study one month post-diagnosis. Site staff will be trained in this process during site initiation visits to ensure that patients are not invited to the study within one month of diagnosis.

Patient eligibility will be confirmed by the study team during monitoring visits by consulting the patient’s medical record, however, if there are any concerns during the researcher visit regarding the patient’s eligibility, they will not be recruited.

Eligible patients may nominate a primary carer, if they have one, to be invited to participate in the study. In this situation, patients’ usual care team, or the patient, will provide the carer with a Carer Information Sheet (CIS) (if appropriate) and ask them to consider their involvement in the study. It is recognised that close family member, friends and partners may or may not view themselves as providing care, and this will be made explicit in the information sheet that this term refers to “those who are close to the patient but who may not be providing hands on, personal care”.

Patients expressing an interest will be asked to contact the research team directly, or give permission for their details to be passed on. Potential participants will then be contacted via telephone by the research team to arrange an appointment. At this time, patients will be asked whether they would like to nominate a carer and if so, a Carer Information Sheet (CIS) will be sent by the study team. Patients/carers will then be visited by the researcher and all participants will be consented in accordance with Good Clinical Practice (GCP), given the opportunity to ask questions and assured that their consent is voluntary and may be withdrawn at any time without giving a reason and without detriment to their care.

Written informed consent will then be taken from patients and carers separately (if appropriate) and baseline assessments completed. Patients and carers may be seen together by the researcher, or separately upon request. Patients will have had at least 24 hours to consider their decision, including time to discuss with family or friends before a researcher visits.

However, if it is only possible to provide a CIS at the researcher visit, carers will be asked to provide written informed consent at the researcher visit. This is justified as patients will already have study information, the study is low risk in terms of intervention and it is important not to take up more time than is necessary on study procedures thus minimising study burden for the patient. However, if carers would prefer to consider their potential involvement for a longer period, then a follow up visit (in person or via the phone) by the researcher would be arranged within seven days.

At the end of the baseline assessment visit, patients will then be directed by a researcher either face to face or via a letter, to arrange a double appointment to be seen either at their GP practice or at home within two weeks. The study team will also contact the relevant practice so an alert can be placed to indicate that the patient has consented to the trial and will require a double appointment. Ongoing assessment of capacity of patients/carers to provide informed consent will take place during follow-up data collection at 1, 3 and 6 months.

Consent is in relation to the provision of data for the study and agreement to arrange a needs assessment appointment as directed by a researcher, as distinct from the consent provided by the practice cluster agreement to take part in the study.

After confirmation of eligibility, informed consent and collection of baseline measures, consented participants will be allocated a unique study identification reference which will be recorded upon a consent form. The research team will then add new participants to a centrally held database, with identifiable and non-identifiable information separate at this point and stored in separate locked facilities, linked by the unique study ID.

**3.3.3 Randomisation**

The unit of randomisation will be the GP practice. Participating surgeries will be randomised (1:1) by the research team to either Arm 1) direct the consenting patient to a GP or nurse to make an appointment in line with usual practice, or Arm 2) direct the consenting patient to a GP or nurse who is known to have completed the NAT:PD-C training. A training log on both arms of the trial will be maintained, the log on Arm 2 will evidence which clinician(s) have received NAT:PD-C training and to whom patients are directed towards. A pragmatic approach to randomising study sites will be adopted. If the interest of a diverse range of GP practices cannot be obtained, a stratified permuted block randomisation will be used to ensure that both arms are balanced according to practice size (small/large). This approach will allow the most effective method of recruiting patients for a NAT:PD-C consultation to be determined. However, if only a relatively homogenous sample of GP practices can be recruited, a simple 1:1 randomisation will be used.

**3.3.4 Interventions**

Arm 1: Promotion and use of the NAT:PD-C with directed clinical encounter with NAT:PD-C trained clinician.

Arm 2: Promotion and use of the NAT:PD-C with directed clinical encounter with a clinician in line with usual practice

All recruited patients will receive a needs assessment consultation, either as a 20 minute (“double”) appointment with the GP, or, as a home visit depending on the clinical situation. The purpose of the study is to determine the most effective way of recruiting patients for a NAT:PD-C consultation to inform a future definitive trial. Following 20 minute patient needs assessment consultations, clinical care will continue as usual, with patients followed up for six months post-informed consent.

**3.3.5 Study assessment schedule**

Assessments and study measures for patients/carers at baseline (all measures and demographics) are repeated at one, three and six months post recruitment/registration. Baseline participant questionnaire completion will take place during a face to face interview with the researcher following consent. Follow up questionnaires at one, three and six months will be collected face to face with the researchers where possible, or with telephone and postal completion permitted when not possible with the method of completion recorded. A researcher will contact the patient’s GP practice prior to making contact regarding further questionnaires to ensure that the patient is alive. Patients and carers will then be contacted by a researcher via telephone regarding follow up questionnaires. In the event we were unable to reach participants over the phone, they would be sent a Patient Carer Reminder letter, inviting them to make contact with the research team. Data collection will be undertaken as close to the stated time points as far as possible. However, for practical reasons data will be collected approximately one week either side of one month post-baseline, and two weeks either side of three and six months post baseline data collection. Patient questionnaires are expected to take 45 minutes to complete. Carer questionnaires are expected to take 20 minutes to complete.

Completed NAT:PD-C assessments will be retrieved from the practice clinical record (patient consent forms include consenting for the research team to access data from their electronic clinical record).

**Schedule of assessments**

**Patients**

|  | **Baseline**  **Visit** | **1 month +/- 1 week**  **Visit / phonecall / postal return** | **3 month +/- 2weeks**  **Visit / phonecall / postal return** | **6 month +/- 2 weeks**  **Visit / phonecall / postal return** |
| --- | --- | --- | --- | --- |
| **Demographic** |  |  |  |  |
| Age, sex, ethnicity, relationship status | X |  |  |  |
| Relationship status, living arrangement, accommodation and postcode | X | X | X | X |
| Cancer type and stage | X |  |  |  |
| Date of cancer diagnosis, current cancer treatments, palliative care input | X | X | X | X |
| Charlson Co-morbidity index | X | X | X | X |
| **Needs assessment consultation** |  |  |  |  |
| Attendance for needs assessment consultation |  | X |  |  |
| Length of consultation |  | X |  |  |
| NAT:PD-C use (yes/no) |  | X |  |  |
| **Unmet needs** |  |  |  |  |
| Supportive Care Needs Survey (SCNS-SF34) | X | X | X | X |
| **Symptoms** |  |  |  |  |
| Revised Edmonton Symptom Assessment System (ESAS-r) | X | X | X | X |
| **Mood and quality of life** |  |  |  |  |
| EORTC QLQ-C15-PAL | X | X | X | X |
| **Performance status** |  |  |  |  |
| Australian modified Karnofsky Scale (AKPS) | X | X | X | X |
| **Health care service utilisation and referral patterns** |  |  |  |  |
| Resource Use Questionnaire | X | X | X | X |
| EQ-5D-5L | X | X | X | X |
| ICECAP Supportive Care Measure (ICECAP-SCM) | X | X | X | X |

**Carers**

|  | **Baseline**  **Visit** | **1 month +/- 1 week**  **Visit / phonecall / postal return** | **3 month +/- 2weeks**  **Visit / phonecall / postal return** | **6 month +/- 2 weeks**  **Visit / phonecall / postal return** |
| --- | --- | --- | --- | --- |
| **Demographic** |  |  |  |  |
| Age, sex, ethnicity, relationship status | X |  |  |  |
| Relationship status, living arrangement, accommodation and postcode | X | X | X | X |
| Palliative care input | X | X | X | X |
| **Needs and ability to provide care** |  |  |  |  |
| Carer Support Needs Assessment Tool (CSNAT) | X | X | X | X |
| **Wellbeing** |  |  |  |  |
| Carer Experience Scale | X | X | X | X |

**Clinicians/practice staff**

|  | **Pre-recruitment** | **2 weeks post-patient recruitment** | **1 month** | **3 month** | **6 month** |
| --- | --- | --- | --- | --- | --- |
| **Screening** |  |  |  |  |  |
| Eligibility assessment (cancer registry) | X |  |  |  |  |
| **Needs assessment consultation** |  |  |  |  |  |
| NAT:PD-C completion |  | X |  |  |  |
| Length of consultation |  | X |  |  |  |
| **Ongoing study procedures** |  |  |  |  |  |
| Assessment of patient/carer status (living or dead) |  | X | X | X | X |
| Patient/carer place of death |  | X | X | X | X |

**3.3.6 Assessment instruments**

1. Unmet needs. The Supportive Care Needs Survey (SCNS-SF34)^[[22]](#endnote-22)^ will be used to be consistent with the initial testing of the NAT:PD-C. The SCNS-SF34 is a valid and reliable 34-item measure assessing cancer patients unmet needs across the following five domains: psychological, health system information, physical and daily activity, patient care and support, and sexuality. Each item is rated on a 5-point scale: 1=not applicable, 2=satisfied, 3=low need, 4= moderate need, and 5=severe need; and unmet need for each domain is defined as a score of ‘4= moderate need’ or ‘5=severe need’ on any item within each domain, with no need defined as a score of ‘1=not applicable’, ‘2=satisfied’, ‘3=low need’.
2. Severity of symptoms. The revised Edmonton Symptom Assessment System (ESAS-r) will be used to measure the severity of nine symptoms common in cancer patients.^[[23]](#endnote-23)^
3. In addition to the SCNS, mood and quality of life (QoL) will be measured using the EORTC QLQ-C15-PAL.^[[24]](#endnote-24)^,^[[25]](#endnote-25)^ The EORTC QLQ-C15-PAL is a questionnaire developed to assess the quality of life of palliative cancer care patients. The Australian study used the EORTC-QLC-C30, but this is shorter and designed for palliative care.xviii The EQ-5D-5L (Five level) is a generic, five-item, health-related quality of life measure that provides the utility values necessary for quality-adjusting survival in economic analyses.^[[26]](#endnote-26)^ The ICECAP Supportive Care Measure (ICECAP-SCM) is a seven-item utility measure which is designed for use at end of life and also enables quality-adjusted life year calculation.^[[27]](#endnote-27)^ Although the EQ-5D-5L is a well-established tool, the ICECAP is potentially better tailored to this patient population. The best “fit” for the definitive study will be assessed by the study team.
4. Performance status. The Australian modified Karnofsky Scale (AKPS) which has been adapted and validated for use in a palliative care population.^[[28]](#endnote-28)^
5. Demographic measures: age, sex, cancer type and stage, treatment history, ethnicity, relationship status, living arrangement and accommodation, household income, post code as well as the Charlson Co-morbidity index.
6. Place of death
7. Health care service utilisation and referral patterns. A bespoke questionnaire (Resource Use Questionnaire) for capturing patient healthcare resource use will be used. Open questions will be incorporated to aid further development of this tool for a definitive trial.
8. The Carer Support Needs Assessment Tool (CSNAT) will be employed to assess both carer ability to care and wellbeing:^[[29]](#endnote-29)^,^[[30]](#endnote-30)^,^[[31]](#endnote-31)^ This is a one page 15 item questionnaire which matches the carer domains on the NAT:PD-C. In addition, the Carer Experience Scale (CES) will also be used as this will provide utilities.^[[32]](#endnote-32)^,^[[33]](#endnote-33)^

**3.3.7 Study feasibility outcomes**

**a) Recruitment**

The feasibility and success of the recruitment strategy will be evaluated by summarising the numbers of:

- GP practices invited to take part and practices recruited
- Patients identified per practice
- Patients invited to take part by the usual care team
- Patients contacting the research team or GP to allow consent for contact by the researcher
- Patients completing a researcher visit
- Consenting and recruited patients
- Consenting carers
- Patients not recruited due to language issues

**b) Uptake and delivery**

- Number and timing of participants first completed NAT:PD-C
- Number and timing of consultations in which the NAT:PD-C was used or not
- Clinician present in the consultation
- Length of appointments when completing the NAT:PD-C
- Completion rates of items within the NAT:PD-C.
- Total participants with completed baseline demographic measures
- Clinical data including place of death, health service utilisation and referral patterns
- Self-reported outcomes by time-point drawn from study questionnaires
- Missing data for self-reported outcomes (at the individual item level and for entire outcomes).
- Proportion of participants successfully followed up through different modes of administration (postal, online, telephone).
- Patient retention including the number of participants withdrawing, and the timing and reasons for withdrawal.

**c) Data collection and quality**

- Questionnaire completion rates
- Amount/pattern of missing data in proposed definitive study primary outcome, the SCNS
- Withdrawals
- Patient/carer outcomes e.g. unmet needs, ability to care, quality of life, performance status, demographics, place of death, health service utilisation and referral patterns.

A traffic light system will be utilised to determine the outcome and criteria for progression of the feasibility trial to the Phase III trial, with respect to: Recruitment; Uptake and delivery; and Data collection and quality. A trial steering committee will be convened to provide external scrutiny of these criteria, whether they are met at the end of the feasibility phase, and whether the trial should progress to Phase III.

Red       Cannot proceed to Phase III

Amber Proceed to Phase III with adaptations to study design, i.e. in terms of the:

- Number of practices
- Implementation and training in the intervention
- Methods of data collection

Green Proceed as planned without adaptation

The criteria are applied to each of our feasibility domains: Recruitment, Uptake and Delivery and Data Collection and Quality.

**Recruitment**

It is anticipated that a minimum of 10-15 patients should be recruited from each general practice over 6 months to demonstrate an acceptable recruitment rate to progress to the recruitment of 62 GP practices for a definitive trial.

Red: <7 patients recruited - Insufficient numbers per practice to proceed without increasing the number of GP practices to ≥72 (more than an extra 3 practices per hub would be required for a revised definitive trial.

Amber: 7 – 10 patients recruited - Sufficient numbers per practice to proceed with changes, increasing the number of GP practices to <72 (an extra 3 practices per hub)

Green: 10 – 15 patients recruited - continue to recruit 62 practices in the Phase III trial

**Uptake and delivery**

Attendance at the initial NAT:PD-C GP appointment within one month post registration (baseline/consent):

Red: <50%

Amber: 50 – 80%

Green: >80%

**Data collection and quality**

Proposed primary outcome measure, the SCNS, follow up completion rate at 3 months (completed: face to face, via telephone or postal return):

Red: <65%

Amber: 65 – 80%

Green: >80%

**3.3.8 Sample size (cRCT)**

Since this is feasibility, a formal sample size calculation has not been performed.i The aim is to recruit four GP practices from within the NHS Hull Clinical Commissioning Group (CCG) and/or surrounding CCGs (e.g. NHS East Riding of Yorkshire CCG). Fifteen participants per practice will be recruited; 60 across 4 practices over a 6 month recruitment period. It is felt that this will be a large enough sample to inform the practicalities of delivering the intervention in patients, recruitment, uptake, and attrition. This should provide sufficient patient outcome data with which to estimate sample size of a definitive trial.^[[34]](#endnote-34)^

The patient-nominated primary carer of all consented patients will be invited to take part in the study. Patients may nominate one primary carer for invitation/inclusion into the study. There is no target sample size for carers and total number of carers consented to the study will be reported as an outcome. In the event that sufficient carers were not recruited in order to obtain rich data for qualitative analysis, this finding would inform the study design of any future definitive trial. Although the high prevalence and value of carers who are children is acknowledged, this population has been excluded as outside the scope of this study. This is due to the different range of needs, commissioned services and social support mechanisms which are in place to support them as carers.^[[35]](#endnote-35)^

**3.4 Qualitative Evaluation methods**

In order to achieve the aims and objectives of the study, an embedded qualitative study will run alongside the cRCT to determine the acceptability of: recruitment methods, training tools, the NAT:PD-C and to identify organisational factors which might influence the willingness or ability of GP practices to adopt the NAT:PD-C.

**3.4.1 Eligibility Criteria**

Inclusion:

- Patients: all patients who have participated in CANAssess:PC who are willing to provide informed consent and able to undergo interview
- Carers: all carers who have participated CANAssess:PC who are willing to provide informed consent and able to undergo interview
- GP Practice staff: GPs, practices nurses and administrative staff in participating practices who are willing to provide informed consent and able to undergo interview/focus group
- Specialist palliative care health professionals: health care professionals who accept referrals from primary care services who are willing to provide informed consent and able to undergo interview/focus group

Exclusion:

- Withdrawn participants who have stated they do not wish to participate further in study procedures

**3.4.2 Sample size**

- A purposive sample of patient participants (and their carers) will be interviewed to explore their views of the trial and factors which affect willingness to remain in the trial and complete study measures. The aim is to include a diversity of experience by including patients (and carers), completers and non-completers of the feasibility follow-up, age, sex and according to higher and lower levels of baseline need across the recruiting sites. Participants will be invited for interview after completion of study procedures or withdrawal. The sample size will be determined by data saturation, willingness of patients/carers to participate and total patients/carers eligible to participate.
- A purposive sample of study site: GPs, practice managers, reception staff, practice nurses and others suggested by the practice will be invited to take part in interview/focus group to explore their views on the acceptability and usefulness of the NAT:PD-C, training materials as well as issues relating to implementation of the tool into general practice. An aim is to conduct a focus group in each participating GP practice with interviews offered to staff members unable to take part in a focus group or who would otherwise prefer to take part in an interview. The sample size will be determined by data saturation, willingness of staff to participate and total staff numbers. Participants will only be able to take part in either a focus group or an interview.
- A purposive sample of palliative care specialists (community, hospice and hospital) will be interviewed to explore their views of how the NAT:PD-C would work in practice. The sample size will be determined by willingness of specialists to take part and data saturation.

**3.4.3 Recruitment and consent**

Patients and carers: Patients and carers will be informed in the relevant feasibility trial information sheets that they will potentially be invited to participate in an interview. Those indicating consent will be drawn from the pool from which a purposive sample of patients (and their carers), will be invited to a short semi-structured interview to explore their views of the trial and factors which affect willingness to remain in the trial and complete study measures. Consent for the qualitative element will be taken separately in order to not raise expectations that all participants will undergo interview. Patients/carers will be given the option that interviews are conducted by the same researcher who undertook data collection during the cRCT or may request that a different researcher conducts the interview.

Participants will be interviewed after completion or withdrawal, therefore a separate Patient/Carer Information Sheet (PCIS) will be provided to potential participants at the conclusion of their involvement in the cRCT. If final questionnaires are completed face to face with participants, then the researcher will provide patients/carers with a study invitation and PCIS, give potential participants the chance to ask questions about the study and invite them to consider their participation. If patients/carers are willing to undergo interview, then informed consent may be taken by the researcher and an interview commenced. This is justified as the researcher will have established a relationship with participants over a period of months and would only take consent and proceed to interview if it was felt to voluntary and the least demanding method of conducting the interview for the patient. Should participants express they would prefer to undergo interview at a different time then this would be arranged. Equally, should the researcher judge that patients/carers were too tired or otherwise unable to undergo interview immediately, then a separate appointment would be made.

If final questionnaires are completed over the phone or returned by post, patients/carers will be invited over to take part over the telephone and/or sent a letter with a PCIS inviting them to contact the research team if they are willing to participate. Patients/carers willing to take part in the study will be contacted by the research team and invited to specify a location for interview. A researcher will then meet the patient/carer at a place of their choosing and all participants will asked to provide written informed consented in accordance with GCP. Oral informed consent will also be taken at the commencement of audio-recorded interview.

GP practice staff: A purposive sample of GPs, practice managers, reception staff and practice nurses from practices participating in the feasibility study will be invited to undergo a short semi-structured interview and/or focus group discussion. Interviews/focus groups will explore participants’ views of the acceptability and usefulness of study training procedures and the NAT:PD-C itself as well as issues relating to implementation. Potential participants will be provided with a PCIS and invited to consider their involvement. A Consent Form will have the options of consenting to interview and/or focus group. However, participants will only be able to participate in terms of **either** an interview **or** a focus group to prevent duplication of data collected. Potential participants will be given the opportunity to ask questions regarding the nature of their involvement in the study. However, as this sample group are to be asked about their professional, rather than personal views and there is low risk of any harm from their involvement, for pragmatic reasons there will be no formal ‘cooling off period’ and potential participants will be free to designate the timing of any interview following consent. Written informed consent will then be taken from willing participants and a) an interview time and date will be designated by the participant, or b) participants will be invited to a focus group session. All recruitment and data collection will be conducted in line with GCP. Interviews/focus groups will take place during implementation of the NAT:PD-C after practices have recruited at least 3 patients and at the conclusion of the study.

Palliative Care Specialists: A purposive sample of palliative care specialists will be interviewed to explore their views of how the NAT:PD-C would work in practice. Potential participants will be identified by sending a study invitation and Specialist Palliative Care Information Sheet (SPCIS) to specialist palliative care services known to receive referrals from primary care practices to a short interview or focus. Potential participants will be invited to contact the study team if they are willing to discuss their involvement in the study. A researcher will then make contact to discuss the study, make arrangements for interview and/or focus group. Written informed consent will be taken prior to commencement of any interview or focus group, with GCP adopted at all stages of study procedures. Interviews/focus groups will take place during implementation of the NAT:PD-C and at the conclusion of the study.

At the conclusion of the study, researchers will check with the GP practice that each study participant is still alive, then all patient/carer participants will be sent a thank you letter. Healthcare professionals will be provided with a certificate of contribution which can be used to demonstrate their Continuous Professional Development (CPD) and will also be sent a Participant Thank You Letter.

**3.4.4 Qualitative data collection**

**Researcher training and piloting**

All researchers will be GCP trained and will undergo training conducted by a palliative care physician (MJ) regarding palliative care issues of patients and carers. This will include guidance on how to appropriately and sensitively aid patients/carers in completing study documents questionnaires. Internal pilot testing will be conducted for all interviews/focus groups to be conducted using an interview schedule/focus group prompt tool.

**Interview procedures**

All interviews will be conducted at a place of the participants’ choice by a researcher who has received DBS certification and GCP training and compliant with the lone worker policy at the University of Hull. Semi-structured interviews will be conducted using an appropriate topic guide developed from relevant literature, team experience and patient/carer representatives to probe areas of interest from different participant groups (patients/carers, GP practice staff, palliative care specialists). Interviews will last approximately 30 minutes and will be audio-recorded, verbatim transcribed and analysed using template analysis and hierarchical coding.

**GP Staff focus groups**

All focus groups will be facilitated by two researchers using a topic guide developed from relevant literature and team experience (including GP input) to probe areas of interest. Focus groups will have a maximum of 10 participants and will be conducted within study sites. Sessions will focus upon two key issues:

1. Appropriateness and usefulness of study training
2. Barriers and facilitators to the implementation of the NAT:PD-C

The aim is to complete one focus group per practice, to explore how the practice team has worked together to implement study procedures and the NAT:PD-C. All practice staff will be invited to focus groups, with those willing but unable to attend a group, but wishing to contribute invited to an individual interview lasting approximately 30 minutes to be invited to interview. Focus groups will last approximately one hour and will be audio-recorded, verbatim transcribed and analysed using template analysis and hierarchical coding.

**4.0 Data analysis**

**4.1 Cluster Randomised Controlled Feasibility Trial: Statistical Analysis Plan (SAP)**

**General considerations**

Statistical analysis will be undertaken by the trial statistician. The analysis plan in this section will be reviewed and a final more detailed analysis plan written, finalised and agreed by wider team prior to analysis.

Statistical analysis will be conducted once the trial is closed to recruitment and six month follow up has been achieved. As this is a feasibility trial of short duration, no interim analysis is warranted.

The flow of individual participants through each stage of the trial will be reported in accordance with the CONSORT 2010 statement extension to pilot and feasibility trials;**Error! Bookmark not defined.** this will include the number of persons evaluated for potential enrolment, randomly assigned to each group, who received treatment as allocated, who completed treatment as allocated, who completed follow-up as planned and included in the main analyses in each group.

The feasibility criteria will be recruitment rate and duration, retention rate, compliance, completion rates and acceptability of the intervention. The recruitment rate, consisting of the eligibility and consent rate will be calculated with 95 % confidence interval (CI).

A table showing baseline demographic and clinical characteristics for each group will be presented to indicate any differences between groups. Patient characteristics will be summarised using appropriate statistics. Medians (range) will be reported for ordinal data, mean (95 % CI) for continuous data and raw count (number, %) will be reported for nominal data.

For the patient and carer outcome data, due to the nature of this feasibility study, no formal statistical tests will be undertaken. Descriptive statistics, mean (standard deviation) for continuous outcomes and raw count (%) for categorical outcomes, will be reported. This will be presented for each group at each time point: baseline, 1 month, 2 month and 3 months. The variability in these measures at both the level of patient and carer and GP practice will be calculated. This will be used to inform the power calculation for the definitive RCT, which will include estimation of an intraclass correlation coefficient.

**4.2 Qualitative process evaluation**

Qualitative analysis of interview and focus group data will be conducted to assess: factors which affect patient/carer willingness to remain in the trial and complete study measures, stakeholder views of views of the acceptability, usefulness of study measures and training, and issues relating to implementation of NAT:PD-C.

All interviews/focus groups will be verbatim transcribed by a study researcher or research administrator or authorised transcriber. All identifiable data will be anonymised. In the event that any digital data was transferred to an external transcriber, data would be anonymised and transferred securely from an encrypted network and a Confidentiality Agreement established between the university of Hull and the external transcribing agency. All transcripts will then be checked for accuracy by a single researcher, with initial emergent areas of interest noted. Transcripts will then be analysed using template analysis.^[[36]](#endnote-36)^ The process starts through initial reading of the transcripts. Transcripts will be coded line by line. Some *a priori* codes may have been identified for themes expected to be relevant by the research team. Where segments of text correspond to *a priori* themes, they are coded as such, however *a priori* codes may be modified or discarded or added to during analysis of the actual data.

Hierarchical coding will begin with broad themes, then encompass successively narrower, more specific ones. Some a priori codes may have been identified for themes expected to be relevant by the research team. Where segments of text correspond to a priori themes, they are coded as such, however a priori codes may be modified or discarded or added to during analysis of the actual data. Hierarchical coding will begin with broad themes, then encompass successively narrower, more specific ones. At least five interview transcripts and two focus groups will be coded by two researchers, but cross-checking for agreement, with emerging themes discussed amongst the study team. Themes will be organised and summarised following analysis of the first few transcripts and a coding ‘template’ will then be developed through comparison of coded transcripts and discussion which will then be applied to the whole dataset and modified in the light of careful consideration of each transcript. Once a final version is defined, and all transcripts have been coded to it, the template serves as the basis for the researchers’ interpretation of the data set and the presentation of findings. This analytical approach will be used ensuring attention to: clarification and justification; procedural rigor; representativeness; interpretive rigour; reflexivity and evaluation rigor; and transferability.

Analysis will be informed by normalisation process theory.^[[37]](#endnote-37)^ This is an analytical approach which focusses upon what participants do as opposed to their attitudes or belief and therefore how behavioural change can most readily be affected. This approach will inform interview and focus group schedules and allow us to appropriately answer questions regarding the practicality and appropriateness of study training, as well has how the NAT:PD-C could work in practice for all stakeholder groups.

**5.0 Data management and confidentiality**

**5.1 Confidentiality**

All participants consented to the study (e.g. patients, carers, GPs etc.) will be assigned a unique anonymised study identification number. All physical copies of identifiable data (e.g. consent forms) will be stored in a locked filing cabinet and kept separately from non-identifiable data (e.g.EQ5D-5L). Identifiable and non-identifiable electronic data will be stored on separate password protected devices.

Consenting participants will be assured of the anonymity of their participation. Following data collection, digital recordings will be transferred to an encrypted device at SEDA as soon as is feasible, with patient identifiable data (e.g. consent form) and non-identifiable data (e.g. questionnaires) stored in separate locked facilities at SEDA. Interviews/focus groups will be transcribed and anonymised as soon as is feasible. Audio recordings will then be destroyed. All participant data will be stored in accordance with the Data Protection Act, 1998.^[[38]](#endnote-38)^

**5.2 Data Management**

The Core Project Team (CPT) will provide set-up and monitoring and oversee: study conduct including randomisation design and implementation, database development and provision, data collection and ongoing data management, study monitoring and statistical analysis and reporting.

Each recruiting GP practice will have a dedicated researcher and administrator who will be responsible for site identification, training, recruitment, data collection from local GP records and ongoing promotion of the feasibility study. There will be clear lines of responsibility for project management, monitoring timescales, recruitment, compliance, analysis, ethical issues, and safety.

Patient/carer self-reported questionnaires will be entered on to a password protected database (Microsoft Access) and checked for accuracy by a second person. Original copies of data collection forms will be stored for the duration of the study within SEDA office premises.

Data will be monitored for quality and completeness by the study team, using established verification, validation and checking processes. Missing data, except individual data items collected via the patient reported questionnaires, will be queried until they are received, confirmed as not available, or when the trial is at analysis.

**5.3 Data monitoring and quality assurance**

Data monitoring and quality assurance reports will be overseen by the Trial Steering Committee (TSC). The TSC will oversee the ethical and GCP conduct of the study as well as study analytical methods. Data cleaning, quality and monitoring will be undertaken by study researchers.

**Study monitoring procedures**

Site set-up training and initiation will be conducted by GCP-trained researchers from the SEDA research group. Sites will be monitored after the first two patients have been recruited and thereafter, triggered by concerns of the research team, or by site request and at the end of the study. Study sites may request additional monitoring and training support throughout the study. Monitoring will focus upon checking whether site study procedures are in accordance with the protocol. For example, data will be retrieved from GP computer systems/patient medical records to confirm the eligibility of participants based upon their clinical data.

The research team will provide set-up and monitoring of study conduct including randomisation design and implementation, database development and provision, protocol development and ethics submissions, data collection and ongoing data management, monitoring schedule and statistical analysis and reporting.

**6.0 Ethical considerations**

Good Clinical Practice (GCP) will be exercised throughout the study. No participant will be approached or recruited prior to the necessary ethical and governance permissions being in place. In the following sections, the ethical considerations of this study are discussed.

**Informed consent**

The overall purpose of this feasibility study is to determine the feasibility of conducting a definitive trial of the NAT:PD-C. The nature of this feasibility study will be made clear to study participants in study information as well as during informed consent in line with guidance.^[[39]](#endnote-39)^

Good practice will be put in place in terms of participants’ capacity to provide informed consent. A Disclosure and Barring Service (DBS) certified, GCP-trained researcher will take informed consent face-to-face only if they are confident that participants have a good understanding of the study purpose and requirements, can weigh up the information and make, and communicate a decision to participate. For patients and carers, if during follow up any information becomes available, either during face to face consultations or by other means (e.g. family member/carer/clinician/patient raises concern), that a participant no longer has the capacity to provide ongoing informed consent then they will be withdrawn from the study by the study team. All data collected up until such a point would be retained.

Although this is a mixed methods study, patients will be consented for the cRCT and qualitative interviews separately. This is because we will not interview every consented patient and therefore wish to avoid raising patient expectations that they will definitely be interviewed.

**Randomisation**

It is feasible that some patient participants may have an existing relationship with a preferred clinician. We acknowledge that through randomisation, patients may be directed towards a clinician whom they do not have an existing relationship with and the potential this raises for unease amongst patients. This possibility will have been made clear to patients prior to taking informed consent. We are attempting to define the best way of recruitment for all patients to receive a NAT:PD-C needs assessment however, all patients will receive a needs assessment whether or not their clinician uses the NAT:PD-C to guide the consultation. Patients will be aware that they are free to withdraw from the study at any point without giving a reason and with no detriment to the level of care they receive.

**Interviews:**

It is noted that because of the nature of palliative care discussions as part of usual clinical care, some patients/carer may become upset during interviews. During interviews, if the researcher has any concerns in this regard then they will offer to pause the interview to allow the patient/carer time, and continue if and when they are happy to do so. If the researcher has serious concerns, then a judgement decision would be exercised to end the interview. The researcher would pass any clinical concerns to the clinical team if necessary.

**6.1 Safety issues**

By entering patient/carer homes, the researcher will be entering into participants’ personal space and lives. This raises a number of issues. Firstly, although it is deemed minimal, there is a risk in terms of the safety of the researcher when entering other peoples’ homes. The researcher will comply with the University of Hull Lone Worker Policy. Secondly, by entering participants’ homes, there is a possibility that the researcher may be witness to inappropriate, illegal or abusive behaviours and activities. In any such circumstances, the researcher will either: seek guidance from the participants’ GP regarding any social issues or call relevant authorities.

**6.2 Potential benefits for participants**

The study may improve identified and managed needs for participating patients by triggering an holistic assessment that they might otherwise not have had. However, this is not proven (hence the need for this study) and the anticipated benefit is to improve the care of people with cancer in the future.

**6.3 Potential risks and burdens to participants**

Completion of study measures has the potential to place a time and emotional burden upon participants. To reduce this potential burden, participants will be given the option of assistance with completing study measures: with a researcher visit, with telephone support for questionnaire completion or in their own time via postal return. Participants will always be allowed to designate the venue of any contact with a researcher (e.g. patient home).

Additionally, some people may be upset when talking about their cancer, care needs, and concerns related to living with cancer. The researcher will be at hand to address any immediate concerns, and will be able to pass these onto the patient’s usual clinical team if required, with the patient’s permission.

Carers:

Caring for a loved one with advanced cancer can be a positive and rewarding experience. However, the act of caring can also be extremely challenging. Carers are known to have higher risk factors than non-carers for psychological, physical, social and financial problems.^[[40]](#endnote-40)^ Moreover, it is recognised that people close to cancer patients may or may not view themselves as providing care and may object to the label of ‘carer’.^[[41]](#endnote-41)^ Although carers will be invited to participate in the study, there is some risk that carers – and particularly those who do not wish to take part in the study - may not welcome the presence of researchers in their homes/place of caring. The autonomy of patients with the capacity to consent to take part in the study will always be respected. However, so too will the wishes of a carer in circumstances where the researcher believes that the continuation of study procedures may place themselves or the patient at risk. In such circumstances the patient would be withdrawn from the study.

**6.4 Potential risks for the study team and health professionals**

This is a low risk study, although any research has the potential to generate risks, with regard to the physical safety and emotional wellbeing of the researcher. Most interactions will be in GP surgery, but here may be some situations where the researcher remains in the patient’s home alone, after the clinician has gone, and for those situations the SEDA (Supportive care, Early Diagnosis and Advanced disease) Lone Researcher Policy will apply. Researchers working on the project will receive regular debriefing sessions. The study will at all times adhere to principles of the Research Governance Framework and Good Clinical Practice.xliii Researchers will be in possession of a research passport for all relevant Clinical Commissioning Groups (CCGs).

In recognition that this study may provoke emotional reactions for the person conducting the research, meetings between the researcher and the PI will include discussion of any distress that the researcher might have experienced during the course of the study. The SEDA Research Group also has an informal ‘buddy system’ for researchers, which offers peer-support if/when needed. If deemed necessary, the University has a counselling service that staff can access free of charge.

**6.5 Risks and benefits of the study**

The study poses minimal risk in addition to usual care to all participant groups. Conducting the study in the manner set out in this protocol is justified for the immediate aim of determining the feasibility of a definitive study to test the validated NAT:PD-C tool. Conducting the study will ensure that any future definitive trial will be financially justified and acceptable to all participant stakeholders, with the potential for the NAT:PD-C to better assist clinicians to identify and distinguish between needs that can be addressed by the usual care team and those which require referral for specialist palliative care.

**6.6 Reimbursements**

Participants will be reimbursed for any reasonable out of pocket expenses (e.g. travel expenses). Patients and carers will be formally thanked for their contribution in a letter upon the conclusion of their involvement in the study. Once our findings are available, key results will be fed back to participants if they wish this. GP practices will be consulted to check that any patient/carer who took part is still alive prior to sending out any correspondence. GP practices will be refunded at NIHR rates for staff time spent implementing study procedures and taking part in focus groups/interviews.

**7.0 Study Management**

Trial supervision will be in line with Medical Research Council (MRC) Good Clinical Practice (GCP) guidelines and include a Core Project Team (CPT), Trial Management Group (TMG), and a Trial Steering Committee (TSC). The trial will be conducted in accordance with Medical Research Council (MRC) guidance on Good Clinical Practice (GCP)^^[[42]](#endnote-42)^^ and in line with a combination of Leeds Clinical Trials Research Unit and Humber and East Yorkshire Standard Operating Procedures (SOPs), in accordance with CONSORT standards, and the NHS Research Governance Framework.^^[[43]](#endnote-43)^^

**7.1 Trial Management Group (TMG)**

The Trial Management Group (TMG) has been appointed to be responsible for overseeing the progress of the study. The TMG will consist of: study investigators, research fellow, research associate, PPI representative(s) and a research administrator. The TMG will meet monthly, either face to face or via teleconference.

**7.2 Core Project Team (CPT)**

The day to day work will be carried out by the research associate under the supervision of the research fellow who will be the project manager. Both researchers will be line managed by and report to MJ. Meetings between the CPT will occur weekly or as often as is deemed necessary to respond to the particular requirements of the study. The project manager will contact each study centre monthly, or as often is as needed, to discuss progress. The CPT will be available for study sites to answer queries and provide guidance at all times during working hours for the duration of the study.

**7.3 Trial Steering Committee (TSC)**

A Trial Steering Committee (TSC) will be appointed including a patient representative and will meet every three to six months or in response to unforeseen events. The TSC will oversee and ensure that the study is being conducted in accordance with GCP and relevant regulations. Thereafter, the TSC will consider study progress including recruitment, adherence to the protocol, participant safety and any other relevant issues arising. The TSC will meet every 6 months.

**7.4 Public Patient Involvement (PPI)**

This protocol has been developed and amended with the involvement of public-patient representatives. The protocol and other study documents have been approved by members of the SEDA PPI group. PPI representatives (patient/carer) will sit on the TSC and the TMG during the trial and encouraged to raise any concerns or suggestions regarding the welfare of participants during the study.

**8.0 Safety reporting**

**8.1 Stopping rules**

As this is a feasibility, low-risk study of short duration, there will be no interim analysis and no stopping rules. The TSC will monitor any emerging issues but no events are anticipated which would cause the study to be stopped.

**8.2 Definitions**

For all sites, adverse and serious adverse events will be recorded and reported in accordance with the ICH GCP and the Research Governance Framework 2005.

This is a low risk study in which data will be collected from patients who have had a needs assessment from a clinician. No drugs or medical devices will be implemented to study participants, other than those used as part of clinical care and the likelihood of harm to study participants due to their involvement in the study is extremely low.

**Expected adverse events**

In this population it is expected that episodes of acute illness, infection, new medical problems

and deterioration of existing medical problems will occur and could result in prolonged

hospitalisation, hospital re-admission, significant or permanent disability or incapacity, or

death. In recognition of this, events fulfilling the definition of a serious adverse event will not be

reported in this study unless the event resulted from administration of any research procedure

and fulfils the definition of a Related and Unexpected Serious Adverse Event (RUSAE).

**Expected SAEs– Standard Reporting**

If research staff become aware that a study participant has died, the clinical research staff will

inform the research team using a standard reporting form. Alternatively, the research team may identify that a patient has died during study monitoring or procedures. As this is expected within the study population, it will not be reported to the main REC and will be recorded as a study outcome.

**Related and Unexpected SAEs – Expedited Reporting**

All Related and Unexpected SAEs occurring from the date of consent until end of research

contact (after completion of all research procedures) will be recorded on the

Related/Unexpected Serious Adverse Event Form. Related and Unexpected SAEs will be identified by the research team during study monitoring and participant contacts.

The study team will be responsible for reporting SAEs in accordance with local HREC requirements to the Research Ethics Committee that gave a favourable opinion of the study and the sponsor (University of Hull) within 15 days of the chief investigator becoming aware of the event using the NRES safety report form available from.

Expedited reporting of events to REC and the Sponsor will be subject to current NRES guidance and Sponsor requirements.

For each Related/Unexpected SAE the following information will be collected:

 date of SAE;

 full details in medical terms with a diagnosis, if possible;

 its duration (start and end dates, and times, if applicable);

 action taken;

 outcome.

**8.3 Progress Reports**

An annual summary report will be submitted to the main REC (UK) which gave the favourable opinion 12 months after the date on which the favourable opinion was given at the end of the study according to the current NRES guidance.

**9.0 Dissemination and archiving**

**9.1 Dissemination plan**

We will seek to publish trial findings in high impact medical journals as well as presenting results at conferences in the UK and internationally. Study findings will also be shared with all participants who indicate during consent that they would like to receive the results of the study.

**9.2 Archiving**

At the conclusion of the study, all study documentation, site files and the trial master file will be stored at the University of Hull for a period of five years. Identifiable and non-identifiable data will be stored in separate locked facilities in appropriate conditions. All digital data will be stored on the secure password protected university server, accessible only by authorised members of the research team.

**10.0 Gantt Chart**

|  | **Time (months)** | | | | | | | | | | | | | | | | | |
| --- | --- | --- | --- | --- | --- | --- | --- | --- | --- | --- | --- | --- | --- | --- | --- | --- | --- | --- |
|  | 2016 | | 2017 | | | | | | | | | | | 2018 | | | | |
| **Task** | **Pre** | Dec | Jan | Feb | Mar | Apr | Jun | Jul | Aug | Sep | Oct | Nov | Dec | Jan | Feb | Mar | Apr | May |
| Electronic NAT development | X | X | X |  |  |  |  |  |  |  |  |  |  |  |  |  |  |  |
| Ethics/MRA approvals | X | X | X | X | X |  |  |  |  |  |  |  |  |  |  |  |  |  |
| Site identification and recruitment |  |  | X | X | X |  |  |  |  |  |  |  |  |  |  |  |  |  |
| Site capacity assessment |  |  |  | X | X |  |  |  |  |  |  |  |  |  |  |  |  |  |
| Site set-up and training |  |  |  | X | X | X |  |  |  |  |  |  |  |  |  |  |  |  |
| cRCT recruitment |  |  |  |  |  | X | X | X | X | X | X |  |  |  |  |  |  |  |
| Qualitative evaluation recruitment |  |  |  |  |  |  | X | X | X | X | X | X |  |  |  |  |  |  |
| Site closure |  |  |  |  |  |  |  |  |  |  | X | X | X |  |  |  |  |  |
| Data entry |  |  |  |  |  | X | X | X | X | X | X | X | X |  |  |  |  |  |
| Data cleaning |  |  |  |  |  | X | X | X | X | X | X | X | X |  |  |  |  |  |
| Analysis |  |  |  |  |  | X | X | X | X | X | X | X | X | X | X | X |  |  |
| Report writing |  |  |  |  |  |  |  |  |  |  |  |  |  |  |  | X | X | X |
| Dissemination |  |  |  |  |  |  |  |  |  |  |  |  |  |  |  | X | X | X |
| TMG meetings |  | X | X | X | X | X | X | X | X | X | X | X | X | X | X | X | X | X |
| TSC meetings |  |  | X |  |  |  |  | X |  |  |  |  |  | X |  |  |  | X |

**10.0 References**

1. Eldridge S, Chan CL, Campbell MJ, Bond CM, Hopewell S, Thabane L, Lancaster GA on behalf of the PAFS consensus group. BMJ, 2016; 355:i5239 [↑](#endnote-ref-1)
2. Stop Cancer Now. Lancet 2013; 9(1381(9865):):426-427. [↑](#endnote-ref-2)
3. Armes J, Crowe M, Colbourne L et al. Patients' supportive care needs beyond the end of cancer treatment: a prospective, longitudinal survey. J Clin Oncol 2009; 27(36):6172-6179. [↑](#endnote-ref-3)
4. Waller A, Girgis A, Lecathelinais C et al. Validity, reliability and clinical feasibility of a Needs Assessment Tool for people with progressive cancer. Psychooncology 2010; 19(7):726-733. [↑](#endnote-ref-4)
5. Waller A, Girgis A, Johnson C et al. Improving outcomes for people with progressive cancer: interrupted time series trial of a needs assessment intervention. J Pain Symptom Manage 2012; 43(3):569-581. [↑](#endnote-ref-5)
6. Quality Health. National Cancer Patient Experience Survey. https://www.quality-health.co.uk/surveys/national-cancer-patient-experience-survey. 2015. 20-6-2015. Ref Type: Electronic Citation [↑](#endnote-ref-6)
7. Funk L, Stajduhar K, Toye C, Aoun S, Grande G, Todd C. Part 2: Home-based family caregiving at the end of life: a comprehensive review of published qualitative research (1998-2008). Palliat Med 2010; 24(6):594-607. [↑](#endnote-ref-7)
8. Stajduhar K, Funk L, Toye C, Grande G, Aoun S, Todd C. Part 1: Home-based family caregiving at the end of life: a comprehensive review of published quantitative research (1998-2008). Palliat Med 2010; 24(6):573-593. [↑](#endnote-ref-8)
9. Carlson LE, Waller A, Mitchell AJ. Screening for distress and unmet needs in patients with cancer: review and recommendations. J Clin Oncol 2012; 30(11):1160-1177. [↑](#endnote-ref-9)
10. Tamburini M, Gangeri L, Brunelli C et al. Assessment of hospitalised cancer patients' needs by the Needs Evaluation Questionnaire. Ann Oncol 2000; 11(1):31-37. [↑](#endnote-ref-10)
11. Davidson PM, Paull G, Introna K et al. Integrated, collaborative palliative care in heart failure: the St. George Heart Failure Service experience 1999-2002. J Cardiovasc Nurs 2004; 19(1):68-75. [↑](#endnote-ref-11)
12. Sanson-Fisher R, Girgis A, Boyes A, Bonevski B, Burton L, Cook P. The unmet supportive care needs of patients with cancer. Supportive Care Review Group. Cancer 2000; 88(1):226-237. [↑](#endnote-ref-12)
13. Deeken JF, Taylor KL, Mangan P, Yabroff KR, Ingham JM. Care for the carers: a review of self-report instruments developed to measure the burden, needs, and quality of life of informal carers. J Pain Symptom Manage 2003; 26(4):922-953. [↑](#endnote-ref-13)
14. Wen KY, Gustafson DH. Needs assessment for cancer patients and their families. Health Qual Life Outcomes 2004; 2:11. [↑](#endnote-ref-14)
15. Girgis A, Johnson C, Currow DC, et al. Palliative Care Needs Assessment Guidelines.The Centre for Health Research & Psycho-oncology. 2006. Newcastle, NSW. Ref Type: Report [↑](#endnote-ref-15)
16. Osse BH, Vernooij-Dassen MJ, de Vree BP, Schade E, Grol RP. Assessment of the need for palliative care as perceived by individual cancer patients and their families: a review of instruments for improving patient participation in palliative care. Cancer 2000; 88(4):900-911. [↑](#endnote-ref-16)
17. NHS Improvements. Rapid review of current service provision following cancer Treatment. http://www.ncsi.org.uk/wp-content/uploads/Rapid_review_of_current_service_provision_following_cancer_treatment.pdf . 2009. 29-5-2015. Ref Type: Electronic Citation [↑](#endnote-ref-17)
18. Waller A, Girgis A, Currow D, Lecathelinais C. Development of the palliative care needs assessment tool (PC-NAT) for use by multi-disciplinary health professionals. Palliat Med 2008; 22(8):956-964. [↑](#endnote-ref-18)
19. Boland JW, Reigada C, Yorke J, Hart SP, Bajwah S, Ross J, Wells A, Papadopolous A, Currow DC, Grande G, MacLeod U and Johnson MJ. The Adaptation, Face, and Content Validation of a Needs Assessment Tool: Progressive Disease for People with Interstital Lung Disease. J Palliat Med. 2016;19(5):549-555. [↑](#endnote-ref-19)
20. Adams E, Boulton M, Rose P et al. Views of cancer care reviews in primary care: a qualitative study. Br J Gen Pract 2011; 61(585):173-182. [↑](#endnote-ref-20)
21. Federation of the Royal College of Physicians of UK. Census of consultant physicians and medical registrars in the UK, 2012. Data and Commentary. https://www.rcplondon.ac.uk/sites/default/files/palliative_medicine_0.pdf. 2014. London, Royal College of Physicians. 22-6-2015.

    Ref Type: Report [↑](#endnote-ref-21)
22. Boyes A, Girgis A, Lecathelinais C. Brief assessment of adult cancer patients' perceived needs: development and validation of the 34-item Supportive Care Needs Survey (SCNS-SF34). J Eval Clin Pract 2009; 15(4):602-606. [↑](#endnote-ref-22)
23. Alberta Health Services. Guidelines for using the revised Edmonton Symtom Assessment System (ESAS-r). 2010.

    http://www.gpscbc.ca/sites/default/files/Edmonton%20Symptom%20Assessment%20Scale%20(ESAS-r)%20guidelines-3.pdf

    Ref Type: Website, accessed30 November, 2016. [↑](#endnote-ref-23)
24. Groenvold M, Petersen MA, Aaronson NK et al. EORTC QLQ-C15-PAL: the new standard in the assessment of health-related quality of life in advanced cancer? Palliat Med 2006; 20(2):59-61. [↑](#endnote-ref-24)
25. Groenvold M, Petersen MA, Aaronson NK et al. The development of the EORTC QLQ-C15-PAL: a shortened questionnaire for cancer patients in palliative care. Eur J Cancer 2006; 42(1):55-64. [↑](#endnote-ref-25)
26. Herdman M, Gudex C, Lloyd A et al. Development and preliminary testing of the new five-level version of EQ-5D (EQ-5D-5L). Qual Life Res 2011; 20(10):1727-1736. [↑](#endnote-ref-26)
27. Sutton EJ, Coast J. Development of a supportive care measure for economic evaluation of end-of-life care using qualitative methods. Palliat Med 2014; 28(2):151-157. [↑](#endnote-ref-27)
28. Abernethy AP, Shelby-James T, Fazekas BS, Woods D, Currow DC. The Australia-modified Karnofsky Performance Status (AKPS) scale: a revised scale for contemporary palliative care clinical practice [ISRCTN81117481]. BMC Palliat Care 2005; 4:7. [↑](#endnote-ref-28)
29. Aoun SM, Grande G, Howting D et al. The Impact of the Carer Support Needs Assessment Tool (CSNAT) in Community Palliative Care Using a Stepped Wedge Cluster Trial. PLoS One 2015; 10(4):e0123012. [↑](#endnote-ref-29)
30. Ewing G, Brundle C, Payne S, Grande G. The Carer Support Needs Assessment Tool (CSNAT) for use in palliative and end-of-life care at home: a validation study. J Pain Symptom Manage 2013; 46(3):395-405. [↑](#endnote-ref-30)
31. Ewing G, Grande G. Development of a Carer Support Needs Assessment Tool (CSNAT) for end-of-life care practice at home: a qualitative study. Palliat Med 2013; 27(3):244-256. [↑](#endnote-ref-31)
32. Al-Janabi H, Flynn TN, Coast J. Estimation of a preference-based carer experience scale. Med Decis Making 2011; 31(3):458-468. [↑](#endnote-ref-32)
33. Goranitis I, Coast J, Al-Janabi H. An investigation into the construct validity of the Carer Experience Scale (CES). Qual Life Res 2014; 23(6):1743-1752. [↑](#endnote-ref-33)
34. Browne S, Dowie A, Mitchell E, Wyke S, Ziebland S, Campbell N, Macleod U. Patients’ needs following colorectal cancer diagnosis: where does primary care fit in? Br JGenPract 2011; DOI: 10.3399/bjgp11X606582 [↑](#endnote-ref-34)
35. UK Government. Young Carers (Needs Assessments) Regulations 2015. 2015. http://www.legislation.gov.uk/uksi/2015/527/pdfs/uksi_20150527_en.pdf.

    Ref Type: Legislation. [↑](#endnote-ref-35)
36. King K. Doing template analysis. In: Symon G, Cassell.C., editors. Qualitative Organizational Research: Core Methods and Current Challenges. London: Sage; 2012. [↑](#endnote-ref-36)
37. Economic and Social Research Council. Normalization Process Theory. 2016.

    http://www.normalizationprocess.org

    Ref Type: Website, accessed 18 November, 2016. [↑](#endnote-ref-37)
38. United Kingdom Government. Data Protection Act. 1998. London: Stationery Office. [↑](#endnote-ref-38)
39. Thabane L, Ma J, Chu R, Cheng J, Ismaila A, Rios LP, Robson R, Thabane R, Giangregorio L and Goldsmith CH. A tutorial on pilot studies: the what, why and how. BMC Medical Research Methodology. 2010; 10:1 DOI: 10.1186/1471-2288-10-1 [↑](#endnote-ref-39)
40. Schulz R, and Beach SR. Caregiving as a Risk Factor for Mortality: The Carer Health Effects Study. JAMA, 1999;282(23):2215-2219. [↑](#endnote-ref-40)
41. Royal College of General Practitioners. Involving and Supporting Carers and Families

    An educational framework and learning resource for GPs and primary care team. 2014.

    http://www.gmc-uk.org/guidance/29827.asp

    Ref Type: Report [↑](#endnote-ref-41)
42. Medical Research Council. MRC guidelines for good clinical practice in clinical trials. 1998.

    http://www.ct-toolkit.ac.uk/routemap/trial-management-and-monitoring

    Ref Type: Report [↑](#endnote-ref-42)
43. Department of Health. Research Governance Framework for Health and Social Care. 2^nd^ Edition. 2005.

    https://www.gov.uk/government/uploads/system/uploads/attachment_data/file/139565/dh_4122427.pdf

    Ref Type: Report [↑](#endnote-ref-43)
