# Supplementary material for: A cluster randomised trial of a Needs Assessment Tool for adult Cancer patients and their carers (NAT-C) in primary care: A feasibility study
Source: PLoS One. 2021 Jan 28;16(1):e0245647. doi: 10.1371/journal.pone.0245647 (PMC7842977; doi:10.1371/journal.pone.0245647)
Supplement: S1 Box — (DOCX) [file pone.0245647.s013.docx]

**S1 Box. Feasibility outcomes, assessment measures and stop/go criteria.**

| 1. Recruitment.    1. GP practices recruited, willing to receive study training and undergo randomisation    2. Patients identified by practice and invited to the study    3. Patients agreeing to and completing a researcher visit    4. Consenting and recruited patients    5. Reasons patients not recruited    6. Consenting carers 2. Uptake and delivery    1. Clinicians undergoing NAT-C training by site    2. Number and timing of consultations in which the NAT-C was used or not    3. Clinician present in the consultation    4. Length of appointments when using the NAT-C    5. Completion rates of items within the NAT-C. 3. Trial procedures and data collection    1. Total participants with completed baseline demographic measures    2. Clinical data including health service utilisation and referral patterns    3. Proportion of participants successfully followed up through different modes of administration (postal, online, telephone).    4. Patient retention including the number of participants withdrawing, and the timing and reasons for withdrawal.    5. Missing data and pattern of missing data for secondary outcomes    6. Self-reported outcomes by time-point drawn from study questionnaires 4. Primary outcome measure   The proposed primary outcome measure for the definitive cRT, the Supportive Care Needs Survey [SCNS-SF34]^[[1]](#endnote-1)^^[[2]](#endnote-2)^^[[3]](#endnote-3)^, is a valid and reliable 34-item measure assessing cancer patients’ unmet needs across five domains: psychological, health system information, physical and daily activity, patient care and support, and sexuality. Items are rated on a 5-point scale: 1=not applicable, 2=satisfied, 3=low need, 4= moderate need, and 5=severe need; with unmet need for each domain defined as moderate or severe need identified on any item within a domain..   1. Secondary outcome measures*   a. Carer support needs (Carer Support Needs Assessment, (CSNAT))^[[4]](#endnote-4)^,  b. Ability to care (Carer Experience Survey [CES])^[[5]](#endnote-5)^,  c. Quality of life (EORTC QLQ-C15-PAL),^[[6]](#endnote-6)^ d. EQ-5D-5L^[[7]](#endnote-7)^, Edmonton Revised Symptom Assessment Scale (ESAS-R)^[[8]](#endnote-8)^, ICECAP-SCM^[[9]](#endnote-9)^,  d. Performance status (Australia-Modified Karnofsky Performance Status [AKPS])^[[10]](#endnote-10)^  e. Co-morbidities (Charlson Co-Morbidity Index (CCI)^[[11]](#endnote-11)^  f. Health service utilisation (Resource Use Questionnaire [RUQ]).  *Secondary outcome measures scored in accordance with scoring and reporting guidance where available. |
| --- |
|  |
| **Stop / go criteria**  **Recruitment**  A minimum of 10-15 patients recruited from each general practice over 6 months to demonstrate an acceptable recruitment rate to progress to the definitive cRT.  Red: <7 patients recruited - Insufficient numbers per practice to proceed without increasing the number of GP practices to ≥72 in the cRT.  Amber: 7 – 10 patients recruited - Sufficient numbers per practice to proceed with changes, some increase to the number of GP practices or length of recruitment required  Green: 10 – 15 patients recruited  **Uptake and delivery**  Attendance at the initial NAT-C GP appointment within one month post registration:  Red: <50%  Amber: 50 – 80%  Green: >80%  **Data collection and quality**  Proposed primary outcome measure, the SCNS, follow up completion rate at 3 months:  Red: <65%  Amber: 65 – 80%  Green: >80% |

1. Boyes A, Girgis A, Lecathelinais C. Brief assessment of adult cancer patients' perceived needs: Development and validation of the 34-item Supportive Care Needs Survey (SCNS-SF34). J Eval Clin Pract. 2009; 15(4):602-606 [↑](#endnote-ref-1)
2. Bonevski B, Sanson-Fisher RW, Girgis A, et al (the Supportive Care Review Group). Evaluation of an instrument to assess the needs of patients with cancer. Cancer. 2000; 88(1):217-25. [↑](#endnote-ref-2)
3. Sanson-Fisher RW, Girgis A, Boyes A, Bonevski B, et al (the Supportive Care Review Group). The unmet need supportive care needs of patients with cancer. Cancer. 2000; 88(1):226-37. [↑](#endnote-ref-3)
4. Ewing G, Brundle C, Payne S, et al. The Carer Support Needs Assessment Tool (CSNAT) for use in palliative and end of life care at home: a validation study. J Pain Symptom Manage. 2013; 46:395–405. [↑](#endnote-ref-4)
5. Al-Janabi H, Flynn T, Coast J. Estimation of a preference based Carer Experience Scale. Med Decis Making. 2011; 31: 458–68. [↑](#endnote-ref-5)
6. Groenvold M, Peterson MA, Aaronson NK, et al. The development of the EORTC QLQ-C15-PAL: a shortened questionnaire for cancer patients in palliative care. Eur J Cancer. 2006; 42(1):55-64. [↑](#endnote-ref-6)
7. Balestroni G and Bertolotti G. EuroQol-5D (EQ-5D): an instrument for measuring quality of life. Monaldi Arch Chest Dis. 2012; 78(3):155-159. [↑](#endnote-ref-7)
8. Watanabe SM, Nekolaichuk C, Beaumont C, et al. A multi-centre comparison of two numerical versions of the Edmonton Symptom Assessment System in palliative care patients. J Pain Symptom Manage. 2011; 41:456-468. [↑](#endnote-ref-8)
9. Huynh E, Coast J, Kinghorn P, et al. Values for the ICECAP-Supportive Care Measure (ICECAP-SCM) for use in economic evaluation at end of life. Soc Scie Med. 2017; 189:114-128. [↑](#endnote-ref-9)
10. Abernethy AP, Shelby-James T, Fazekas BS, et al. The Australia-modified Karnofsky Performance Status (AKPS) scale: a revised scale for contemporary palliative care clinical practice [ISRCTN81117481]. BMC Palliat Care. 2005; 4:7. [↑](#endnote-ref-10)
11. Charlson ME, Pompei P, Ales KL and MacKenzie CR. A new method of classifying prognostic comorbidity in longitudinal studies: development and validation. J Chronic Dis. 1987; 40(5):373-83. [↑](#endnote-ref-11)
