# Supplementary material for: A cluster randomised trial of a Needs Assessment Tool for adult Cancer patients and their carers (NAT-C) in primary care: A feasibility study
Source: PLoS One. 2021 Jan 28;16(1):e0245647. doi: 10.1371/journal.pone.0245647 (PMC7842977; doi:10.1371/journal.pone.0245647)
Supplement: Attachment — Submitted filename: Response to editor and reviewer comments.docx [file pone.0245647.s014.docx]

|  | Reviewer 1 |  |
| --- | --- | --- |
| 1 | The authors stated that “After slow initial recruitment, inclusion criteria were amended to include patients with active cancer irrespective of treatment intent. A further amendment allowed inclusion of a pre-paid Expression of Interest Form (EOI) with study invitations and patient information.” Please provide the quantitative measurements of the recruitment rate before and after each amendment and comment on the rationale of these amendments. | Thank you, we have added recruitment rates for before and after the amendment.  “During September and October 2017, 0 patients were recruited (0 per month). Following an amendment which broadened the inclusion criteria to include any patient with active cancer regardless of prognosis and allow inclusion of EOI forms with study invitations, early recruitment problems were solved. Between November 2017 and the end of March 2018, 47 patients were recruited (11 patients a month).”  Additionally, during dealing with this point, we noticed that we have incorrectly reported that the alteration of the inclusion criteria and change to allow Expressions of Interest form were reported as two separate amendments, when actually this was a single amendment. We have amended this within the ‘patient/carer recruitment’ section  “After slow initial recruitment, inclusion criteria were amended to include patients with active cancer irrespective of treatment intent and allow inclusion of a pre-paid Expression of Interest Form (EOI) with study invitations and patient information.” |
| 2 | The sample size justification is superficial. The authors should provide the quantitative adjustments, e.g., the precision analysis, in addition to a reference. | Thank you. We have added the following text and references :  “As this was a feasibility study, a formal calculation was not appropriate as effectiveness was not being assessed [11]. Sample size recommendations for feasibility and pilot studies vary but numbers between 12 and 50 seem to be acceptable to address variation around baseline measures [12]. ”  11. Lancaster GA, Dodd S, Williamson PR. Design and analysis of pilot studies. recommendations for good practice. J Eval Clin Practice 2004;10:307-312  12. Julious SA. Sample size of 12 per group rule of thumb for a pilot study. Pharm Stat 2005;4:287-291 |
| 3 | The authors stated that “the mean response was used to impute missing item(s)” (line 171). This is not recommended because the variation is underestimated. Please use the multiple imputation approach for missing data analysis. | Thank you, our imputation method is consistent with that recommended by the user guide for our primary outcome the Supportive Care Needs Survey and used the individual participants mean response where at least 50% of questions were answered within a domain. No imputation of missing data was otherwise made. The manuscript reads “Where ≥50% of questions were answered within a domain, the mean response was used to impute missing item(s). Otherwise, individual domain scores were missing.” We have therefore not made these suggested changes, which would also not materially alter findings.  McElduff P, Boyes A, Zucca A and Girgis A. Supportive Care Needs Survey: A guide to administration, scoring and analysis. 2004. Available from: [www.researchgate.net/publication/239565229](http://www.researchgate.net/publication/239565229) |
| 4 | In addition to reporting the mean and SD, the authors should also report the 95% confidence interval for the outcome measurements. | Thank you. We have now added 95% confidence intervals for all outcome measures (Table 3 and S:5) |
| 5 | The authors should condense the article to improve the readability. | Thank you – we do not feel we can further condense the article because we are i) reporting a mixed-methods feasibility study with both quantitative and qualitative data, and ii) reporting against CONSORT recommended guidance for such a study design. Please also see response to comment 6 (from Reviewer 2) |
|  |  |  |
|  | Reviewer 2 |  |
| 6 | This paper reports on a feasibility study of an intervention incorporating the NAT-C tool that targeted patients with cancer and was undertaken in preparation for a definitive trial evaluation. It’s an interesting topic. However, I found the manuscript difficult to follow in parts. As per comments below, I think more background information is needed as well as a clearer overview of what the intervention entailed.  I would encourage the authors to also consider the use of a reporting guideline for reporting randomised pilot and feasibility studies conducted for the overall paper (https://www.equator-network.org/reporting-guidelines/consort-2010-statement-extension-to-randomised-pilot-and-feasibility-trials/ ) as well as for the intervention (https://www.bmj.com/content/348/bmj.g1687) | Thank you for these comments.   1. We agree that the article is very dense. We feel this is inevitable as a mixed-methods report of a complex intervention within the word limits of the Journal. We have reviewed the text to see if we could better signpost to help with navigation – however, taking into account your later comment about there being a lot of sub-headings, we are loathe to introduce any more. Unless the Editor feels we should re-structure, we would prefer not to risk further muddying the waters. We hope the way we have responded to other comments will help address this concern. 2. We have now reported further background information in the introduction (see below)   “Many people with cancer and their carers have physical, psychological, emotional and social problems, which commonly go unidentified or addressed. Unmet needs in patients with cancer and their carers are common but poorly identified and addressed [1]. One consequence of this, is that access to palliative care and other supportive services is often based upon prognosis, rather than present need and that many people who might benefit from such services, do not have access to them [2]. Early identification of patient and carer problems in primary care may help improve access to palliative care and other supportive services and improve the quality of life of cancer patients and their carers.  One way in which primary care is responsive to the needs of cancer patients, is through the provision of a Cancer Care Review (CCR) as part of the Quality and Outcome Framework [3]. A CCR aims to help the person affected by cancer to understand what information and support is available to them locally. GP practices receive Quality and Outcomes Framework payments for conducting cancer reviews within six months of patients’ diagnosis. However, there is no guidance regarding what a cancer review should involve or evidence-based template available.   3. We have added some more details to describe the intervention in the introduction. Please note, due to our ongoing main trial, we are reluctant to provide too much detail regarding the intervention due to risk of contamination between the intervention arm and the usual care arm. We append a TiDIER checklist to demonstrate adequate description of the intervention and added text to show we have done this.   “The NAT-C is a structured form, comprising five sections: Priority referral for further assessment, Patient wellbeing, Ability of carer or family to care for patient, Carer/Family wellbeing and Resulting referrals (if required).”  “the NAT-C is described in accordance with items of the Template for Intervention Description and Replication (TIDieR) checklist”   4. Thank you for your suggestion that we use the CONSORT guidance – we have already done so, noted just prior to our results and previously attached (both pilot and cluster extensions) |
| 7 | Title  I find the reference to two study designs in the title confusing  There is no mention of palliative care in the title even though the abstract explicitly mentions palliative care | CONSORT extensions for cluster and feasibility trials recommend we report study designs as such. We have amended the title to illustrate the population and amended reference to palliative care in the abstract.  “A cluster randomised trial of a Needs Assessment Tool for adult Cancer patients and their carers (NAT-C) in primary care: a feasibility study” |
| 8 | Abstract: this would benefit from further review/refinement. I appreciate the challenge in writing an abstract but some of the methods subsection doesn’t give much insight. The following sentences are particularly vague: “Study questionnaires at baseline and 1, 3 and 6 months. Participants booked a 20 minute study appointment post-baseline. Patients, carers and GP practice staff were invited to interviews/focus groups.” For example, there is no indication of what the study questionnaire or post baseline appointment involved. The target patient population needs to be more clearly specified ; “patients with active cancer” is not terribly informative (it doesnt event differentiate between adults and children). There is no clear sense of what the intervention was from the abstract | Thank you, we apologise if the abstract wasn’t previously clear. We have now re-written this almost entirely, making clear   - Adult patient population - That patients attend for a needs assessment - Clarified purpose of interview and focus groups - Clarified purpose of intervention   Our revised abstract also addresses point raised in #12 – we have provided clearer results to support our conclusion |
| 9 | Wording of NAT-C differs from title above | Amended |
| 10 | Results subsection: “Fifteen patient interviews and focus group at each GP practice.” – not a complete sentence and not sure what this is adding | We have amended this sentence as below, but believe it is important to state how many interviews and focus groups were conducted.  “Fifteen patient interviews across the whole study and one focus group at each GP practice were conducted.” |
| 11 | “Feasibility outcomes informed the design of a 2-armed cluster randomised controlled trial to test the effectiveness and cost-effectiveness of the NAT-C compared with usual care.” Is this a direct result of the study? It sounds more like the next step.  Feasibility outcome have not been outlined in the methods subsection | See response to point 8 which also refers to the abstract |
| 12 | Conclusion: I don’t think the relevant results have been outlined to support this; there is also no mention of refinements until this last sentence which somewhat jars with the previous statement that participants found measures acceptable | See response to point 8 which also refers to the abstract |
| 13 | Introduction  General comments: The introduction doesn’t adequately set the scene; the opening two paragraphs don’t provide enough relevant background and instead most of it discusses plans for a future trial. The structure of the introduction could also benefit from refinement; it should flow into a final paragraph that outlines the aim and objectives but at present these seem to be split across the third and fourth paragraphs; the outline of objectives in both of these paragraphs is confusing. I would encourage the authors to consider the Medical Research Council’s framework on the development/evaluation of complex interventions and think about describing the intervention (including how it has been developed) and then working in the need for preliminary testing before a more definitive evaluation. | Thank you, we have substantially revised our introduction in line with comments from all reviewers.  The introduction now emphasises the proposed rationale for the study, highlighting known problems in terms of the delivery of cancer care.  Whilst the aim of the feasibility was to determine prospects for a definitive trial, we have removed information regarding what a future definitive trial may look like from the introduction.  We have also added further description of the NAT-C and emphasised the need for feasibility work in line with Medical Research Council guidance ahead of definitive evaluation. |
| 14 | Specific comments  A stronger opening paragraph is needed. The opening sentence is particularly vague – unmet needs could refer to anything. This needs to set the scene in terms of the scale of the problem that needs to be addressed so that you can build up to outlining specific gaps that this research is addressing later in the introduction | Thank you for raising this. We have re-written the opening paragraph to make clear the types of need which cancer patients may have and outlining the scale of the problem:  “Many people with cancer and their carers have physical, psychological, emotional and social problems, which commonly go unidentified or addressed. Unmet needs in patients with cancer and their carers are common but poorly identified and addressed [1]. One consequence of this, is that access to palliative care and other supportive services is often based upon prognosis, rather than present need and that many people who might benefit from such services, do not have access to them [2]. Early identification of patient and carer problems in primary care may help improve access to palliative care and other supportive services and improve the quality of life of cancer patients and their carers.”  “The NAT-C is a structured form, comprising five sections: Priority referral for further assessment (highlighting any need for palliative care referral), Patient wellbeing, Ability of carer or family to care for patient, Carer/Family wellbeing and Resulting referrals (if required).” |
| 15 | “triage tailored action and audit and service planning” what does this mean/entail | We have amended as follows for clarity:  “helps triage tailored action to address patient concerns, and provides data for use in audit and service planning “ |
| 16 | From paragraph 2, I have no real sense of what the tool is or consists of. From a clinical perspective, the name of the tool is quite non-specific (apart from referring to cancer) and does not give any clear indication that it might link to palliative care | Thank you, see additional info added in response to comment 14.  The NAT-C was originally developed named as the NAT-Progressive disease in cancer (NAT:PD-C). However during adaptation for use in UK primary care, the primary care participants felt strongly that it applied across the whole disease trajectory, and in particular, by taking out “progressive disease” would facilitate early involvement of palliative care according to need rather than prognostic estimates. The form clearly raises the issue of palliative care referral, and we have now added that detail - see response to 16. |
| 17 | Are there any references to back up the points about the tool made in paragraph 3? | Thank you, the point referred to regards our hypothesis – evidence from this feasibility study and potentially the definitive trial ma support the suggestion that the NAT-C *may* help reduce unmet needs. |
| 18 | This is the first time “supportive and palliative care needs” is mentioned; the target population needs to be outlined more clearly and whether this is about providing supportive/palliative care from the point of initial diagnosis or whether it’s more focused on advanced disease and end of life | Thank you. We have clarified this to illustrate that palliative and supportive care needs may be present from diagnosis and to introduce the idea of needs-based access to services.  “Supportive and palliative care needs may be present from diagnosis across multiple domains, including: physical, psychological, emotional, or financial problems. However, Unmet needs in patients with cancer and their carers are common but poorly identified and addressed [1]. access to palliative care and other supportive services is often based upon prognosis, rather than present need and that many people who might benefit from such services, do not have access to them [2]. Early identification of patient and carer problems in primary care may help improve needs-based access to palliative care and other supportive services and improve the quality of life of cancer patients and their carers.” |
| 19 | “by supporting clinicians to systematically assess patient/carer problems across multiple domains” what are these domains that you are referring to | Thank you, we have clarified potential domains of need in response above. |
| 20 | What do you mean by phase 3 in the context of a clinical intervention? See point regarding MRC framework above | Thank you – the terminology used in the MRC Framework for Complex Interventions is an evaluation phase trial – however, the language of phase 3 is more commonly understand which is why we used it. We have now defined this in both terms for the first use of “Phase 3”:  “…before conducting a Phase III (an MRC Framework for Complex Interventions evaluation phase trial)” |
| 21 | Not sure why objectives for a future study are being presented; suggest keeping the focus on the study that is being presented | Thank you, we have removed the objectives of a Phase III study an instead emphasised the importance of feasibility work ahead of an effectiveness trial – in line with MRC guidance on complex interventions.  “The NAT-C may reduce unmet supportive and palliative care needs of cancer patients and their carers by supporting clinicians to systematically assess patient/carer problems across multiple domains and take action. Identified problems may be managed in primary care or through referral to specialist services. In line with Medical Research Council (MRC) guidance, before conducting a Phase III to test the effectiveness of the NAT-C, we needed to address a number of uncertainties in relation to the feasibility of such. This report, therefore, presents the findings of a feasibility cluster Randomised Trial.” |
| 22 | Paragraph 4;  Objective 1 is actually two objectives – recruitment and training  Objective 2; I don’t understand the link between recruitment rate and method of delivery  Objective 3; I don’t understand this objective  Objective 4; what does uptake mean?  Objective 5; is this more about data collection measures? | Thank you, we have amended objective 1 into 2 objectives  Obj3 – Wording has been clarified as below  Obj4 – ‘uptake’ has been changed to ‘use’ to clarify  Obj 5 – Wording clarified as below  Our objectives now read:  “Our objectives were to evaluate: 1) feasibility of recruiting general practitioner (GP [family doctors]) practices; 2) feasibility of training clinicians to use the NAT-C; 3) method of ensuring delivery of a NAT-C appointment in relation to recruitment rate of patients (and carers) and practice preference; 4) proportion of patients unmet patient needs at baseline to investigate rationale for a definitive trial; 5) uptake of the NAT-C by clinicians; 6) completion rates of patient questionnaires; and 7) acceptability of study measures and processes.” |
| 23 | There is no clear mention of fidelity even though is reported later in the results | Thank you – this has was already included within the analysis section  “Intervention fidelity was assessed by reporting completed rates of NAT-C ‘expected items’ (unless irrelevant, for example, if a patient has no carer).” |
| 24 | The final sentence regarding stop/go criteria seems more relevant to methods than introduction | Thank you, this has been moved to the Methods section under ‘study design’ |
| 25 | “The Cancer Needs Assessment (CANASSESS) study was a non-blinded, parallel non-controlled, feasibility trial with 82 cluster randomisation to method of delivery of the NAT-C, qualitative sub-study and parallel process evaluation, in four General Practices.” – very long sentence | Thank you, we have split this into two sentences as below:  “The Cancer Needs Assessment (CANASSESS) study was a non-blinded, parallel non-controlled, feasibility trial with cluster randomisation in primary care. A qualitative sub-study and process evaluation was also conducted in parallel to the feasibility trial.” |
| 26 | Why wasn’t a usual care group included? Surely this would be critical to a future trial? | Thank you for the query. During the design of the study, we felt that the question of whether patient participants would attend a consultation with a GP/practitioner they did not know (although personal lists are being eroded in primary care, they do persist in many practices) was critical to answer – and could seriously derail fidelity to intervention if this was a problem. Therefore the randomisation was with this regard.  The current phase 3 evaluation trial (planned alongside this feasibility trial) has an embedded pilot phase to test willingness of practices to be randomised and patient participants to provide data in both active and control arm practices. |
| 27 | Participants and setting; more information needed about where practices were based; how they were identified, contacted, recruited etc | Thank you, we have added the following information:  “We aimed to recruit four general practices within Hull, East Ridings and North Lincolnshire Clinical Commissioning Groups (CCGs). An Expression of Interest (EOI) request was administered to general practices in these regions by the Clinical Research Network (CRN), after which interested practices contacted the research team directly. We approached interested practices and entered discussions regarding participation in the order in which practices expressed interest.” |
| 28 | “Randomisation was with regard to the method of delivery of the NAT-C” this is the first mention of this being able to be delivered in different ways. Goes back to previous comments about intro not setting the scene adequately | We apologise if this was unclear. We have improved description of this within our objectives in the introduction and clarified the queried phrase within our methods:  “method of delivery ensuring delivery of a NAT-C appointment in relation to recruitment rate of patients (and carers) and practice preference  “Randomisation was to the method of delivery of the NAT-C appointment: patient attendance with a clinician they may not have met before, *versus* a known clinician; to test uncertainty regarding pragmatism versus optimising uptake of the NAT-C guided appointment.” |
| 29 | “test uncertainty on two counts” what does this mean?? | We apologise if this was not clear, see point 28 for amendments. |
| 30 | “Eligible patients were consenting adults with incurable cancer” first clear mention that the focus is on terminal patients; this makes me query the rationale for focusing on general practice – would these patients not be linked to palliative care services?? This should be teased out in the introduction | Our revised introduction now emphasises that unmet needs relating to cancer may be present from diagnosis, but are poorly identified – including for patients with incurable cancer. If patients were receiving palliative care we would expect this to reduce unmet needs and would reduce the rationale for an intervention in primary care. However, given the presence of 72% patients of unmet needs – regardless of whether they are receiving palliative care – this supports the rationale for an intervention in primary care.  We have also added that “eligible patients were initially consenting adults with incurable cancer” ahead of explaining the study amendment and alternation of the inclusion criteria. |
| 31 | “After slow initial recruitment” how long had the study been underway when this change was implemented? This is an interesting feasibility finding and links back to my previous comment above | Thank you. Amendments to a study protocol are to be expected with feasibility work. We have written our manuscript to show both how the trial was initially conceived and changes which were made during the study. Therefore, we note early slow recruitment here, but provide specific dates regarding amendments and their consequences in the results, where dates are provided.  “During September and October 2017, 0 patients were recruited (0 per month). Following an amendment which broadened the inclusion criteria to include any patient with active cancer regardless of prognosis and allow inclusion of EOI forms with study invitations, early recruitment problems were solved. Between November 2017 and the end of March 2018, 47 patients were recruited (11 patients a month).” |
| 32 | I would also like to see dates worked in somewhere within the methods of when the study formally started and finished | We have clarified this in our results section  “The study took place between January 2017 and September 2018.” |
| 33 | The intervention is core to this study yet it is quite poorly described. I have no real sense of what issues were focused on and how GPs were expected to address them – particularly in cases where issues/challenges might have been outsider of their scope of practice etc. There is so much emphasis now on providing sufficient information to enable replication – please consider the use of a reporting guideline such as TiDiER | Thank you, we are sorry if this is unclear. As per response to point 6, we have now included a TiDIER checklist. During feasibility work, we were not testing any kind of causal assumptions and therefore did not apply the TiDIER checklist (which we will do for a definitive trial).  Fidelity to the NAT-C is reported quantitatively (in terms of item completion), more importantly for this feasibility work, clinician attitudes towards the NAT-C are reported within our qualitative findings. |
| 34 | Study assessments and outcomes; abbreviations not defined when first used – same with NoMAD; they would also benefit from some elaboration | Thank you. We apologise, full names are included in Box 1 but we have ensured that all measures are spelt out in full ahead of use of acronyms in the text.  The NoMAD survey is already explained in the Process Evaluation section, the SNCS (proposed primary outcome measure) is also described in Box 1.  A one sentence description is also in Box 1 for secondary outcome measures, we have amended the title of the section to indicate that the description is of what we seek to measure:  “Secondary outcome measures assessing:” |
| 35 | “Feasibility outcomes, assessment measures and stop/go criteria are presented in Box 1.” I’d like to see an outline of key feasibility outcomes listed in the main text. The primary outcome and the tool being used to assess it needs to be outlined in the main text – particularly in terms of choice and suitability | Our revised feasibility objectives are now presented in the introduction. All feasibility outcomes are presented in Box 1. For this feasibility study, there was no single primary outcome. To avoid confusion, we have amended sections in Box 1 to indicate that primary and secondary outcome measures are in fact ‘proposed’ primary and secondary outcome measures.  Given the word limit constraints we feel this is the most “word-efficient” way to present this information |
| 36 | Did participants receive any incentives? | No. |
| 37 | Ethics approval and consent to participate: Unless there is specific journal requirements around this content; I think this could be condensed | Thank you, however, our statement has been prepared to adhere to journal standards. |
| 38 | Results  I think the dates would be better placed in methods | Thank you, we have added recruitment rates in relation to these dates and have therefore left dates within results. |
| 39 | Overall this section is long and a little hard to follow in places – I’d like to see the reported findings more clearly linked to the study objectives/feasibility questions. At present there are just too many subheadings for the reader to navigate. There may also be opportunity for condensing some the information which would enable the introduction and discussion to be teased out more | Thank you for this comment. We have done our best so summarise succinctly but comprehensively with regard to the CONSORT requirements, the high amount of outcomes which emerge from feasibility work – including the NPT study. On balance, we feel that headings serve to streamline the results section, as opposed for need for additional descriptive prose. Please also see response to point 6.  However, we have provided greater clarity within the section by linking important findings to our feasibility objectives, which serves to contextualise findings in terms of gaps and uncertainties presented in the introduction and discussion.  “Five GP practices were approached and four recruited [Figure 1] meeting our feasibility objective.”  “Of all practice staff, 24/33 (73%) GPs and 5/27 (19%) nurses were trained [Table 2], which met our feasibility objective regarding clinician willingness to be trained”  “More people received a NAT-C appointment when directed towards a known NAT-C clinician, informing trial design of a definitive study.”  “At baseline, 34/47 (72%) participants reported at least one moderate to severe unmet need confirming rationale for the study.”  “Overall uptake and delivery of the NAT-C was reported for 32/47 (68%) patient-participants and demonstrated willingness of clinicians to use the NAT-C”  “There was high item completion for the proposed primary outcome measure, the SCNS-SF34 (99.9%, 98.1%, and 95.6% at baseline, 1, 3 and 6 months) supporting use of the SCNS as primary outcome in a definitive trial”  Findings related to our final objective regarding acceptability of study measures are already made clear within our qualitative results section. |
| 40 | “Three main themes were generated. Here, we present the theme which informs our feasibility outcomes: Information and Research.” I don’t follow this | Thank you. Interviews with patients revealed information which went beyond our feasibility objectives and are therefore not reported within the manuscript. For clarity, we have re-phrased as follows:  “A single theme was developed which informs our feasibility outcomes: Information and Research. |
| 41 | I don’t know why quotes are presented with their own separate headings | We have removed the heading to prevent confusion |
| 42 | There seems to be very little focus on the patient interview findings in the results presented | Thank you, there is already a full section on patient views arising within interviews and 1/3 quotes is from a patient. We are satisfied that results as presented reflect patient views regarding our feasibility outcomes. |
| 43 | Discussion  A stronger and more comprehensive discussion is needed that places findings in context of relevant literature. Structure/flow needs refining; no opening paragraph, just a single sentence and then focuses on future work; focus on discussing the work presented and build up to how the findings support you progressing to the next stage of evaluation. There are other cases where single sentences are presented instead of complete paragraphs | Thank you for this comment.  Given the nature of feasibility work, our data do not sit naturally within broader literature regarding the rationale for the trial or the problem in primary care itself – instead the implications of our work are broadly methodological, in context of a future definitive trial.  However, we have undertaken restructuring of our discussion, to put implications of our findings relevant to statements made in the introduction more up front, ahead of evidence-based solutions we have identified for a definitive trial.  We have also added a statement to our introduction, to contextualise discussion points regarding time-delays in primary care trials.  “Evidence-based cancer interventions are needed to improve holistic cancer care in primary care. However, delivery of clinical trials in primary care I known to be challenging, many struggle recruit to sample size and time-delays are common.”  We have also re-read and edited the entire discussion (as well as the rest of the manuscript) for readability. |
| 44 | I would also contest your opening statement as you didn’t succeed in recruiting the specific group of patients that you had originally set out to recruit | Thank you, we have modified this claim:  “Our findings suggest that with the alterations to trial design made during feasibility work, that a multi-centre cluster Randomised Controlled Trial (cRCT) to test the effectiveness of the NAT-C compared to usual care is feasible.” |
| 45 | “The high prevalence, and that we cannot apportion causality to the intervention in this non controlled trial supports a definitive trial.” I don’t know if I agree with the first point regarding prevalence – the study wasn’t designed to assess this; I don’t understand the logic/rationale behind the second point regarding causality | We would suggest that 72% of patients with unmet needs at baseline is does represent high prevalence within our patient group. However, we have also amended the sentence referenced for clarity, to indicate that we are not suggesting that the NAT-C *caused* improvements, only that there were improvements in context of the NAT-C.  Data demonstrate a high prevalence of unmet patient needs at baseline. This study was not designed to demonstrate effectiveness of the intervention, but the high prevalence, uptake of the NAT-C, successful follow-up and completion of outcomes, and patient interviews support the need for a definitive trial. |
| 46 | “Primary care has a major role to play in palliative care [19].” Again goes back to issues flagged with introduction and no detailed explanation of rationale for focusing on general practice in this study; also this isn’t a very informative statement | Thank you for this comment. As per response to point 14, the introduction has been amended to focus on the rationale for the study in general practice.  We have also amended the sentence indicated, to link it to our findings:  “Findings from our process evaluation that 89% of clinicians see the importance of the NAT-C for their work support the suggestion that primary care has a major role to play in palliative care [19].” |
| 47 | “Challenges during feasibility were consistent with other primary care clinical trials; 56% with time extensions and 18% with altered recruitment methods [20]. Use of multiple methods and process evaluation has allowed us to use data driven solutions, to refine the study design of a definitive trial, to maximise prospects of delivery of the study.” I’d like to see more specific discussion of your study findings and them then being placed in context of the literature | See response to comment #43 |
| 48 | “GP practices receive Quality and Outcomes Framework payments for conducting cancer reviews within six months of patients’ diagnosis. However, there is no guidance regarding what a cancer review should involve or evidence-based template available.” This needs to be introduced much much earlier in the paper! | Thank you, this has been added to the introduction |
| 49 | “By broadening the inclusion criteria to all those with active cancer, if effective, the NAT-C could be a validated and tested way of conducting cancer care reviews. It may also inform a consistent, evidence-based approach to referring cancer patients for specialist palliative care input [21]. Referral would be needs-based rather than prognosis-based facilitating earlier integration of palliative care and improve clinical outcomes [22, 23].” - I’d be interest in the authors' view about why they were unable to recruit the intended patient population and what could be done about this? it seems to me that there is a risk that by changing the patient inclusion criteria the intervention still doesn’t reach the original target group (i.e. those with a terminal diagnosis ) | Thank you for this point, we have added the following text to address this issue:  “Early recruitment problems were resolved by broadening our inclusion criteria from patients ‘with incurable cancer’, to patients with ‘active cancer’ and the addition of an EOI form with study invitations. Our patient sample included 9/47 (19%) of participants with metastatic cancer. This suggests that our patient identification methods were appropriate and that people with terminal cancer were willing to take part and that it was the alteration of the study approach which improved recruitment.” |
| 50 | “Feasibility work is an essential part of trial preparation, in particular for trials of complex interventions [24].” Again; more relevant to setting the scene in introduction not the tail end of the discussion | Thank you, we hope that the changes we have made to our introduction and discussion now better contextualise this point as an appropriate concluding remark. Our findings and data-driven solutions reported for a definitive study really do emphasise the usefulness of feasibility work. |
| 51 | Conclusion  A more cohesive/comprehensive concluding paragraph is needed; I don’t think that you can say in advance that a definitive trial is feasible (you’ll only find that out once you get it underway) - need to focus on the feasibility questions that this study has answered and link that to whether it supports progressing to a definitive evaluation | Thank you. We have strengthened the conclusion to reflect the answers to our feasibility questions and moderated our claim that a definitive trial is feasible as below:  “Our feasibility study identified a high proportion of people with cancer with unmet supportive and palliative care needs. This supports the rationale for a definitive study of the NAT-C, a needs assessment tool which encourages systematic identification of patient problems and prompts action. Cancer patients and clinicians support the rationale for the trial and were willing to undergo study measures and deliver the NAT-C respectively. Our findings suggest that a definitive trial of the NAT-C in primary care is feasible, with appropriate refinement, in terms of GP practice and patient participant recruitment, data quality and acceptability and use of the NAT-C. |
